# The Role of Maternal Weight in the Hierarchy of Macrosomia Predictors; Overall Effect of Analysis of Three Prediction Indicators

**DOI:** 10.3390/nu13030801

**Published:** 2021-02-28

**Authors:** Małgorzata Lewandowska

**Affiliations:** 1 Medical Faculty, Lazarski University, 02-662 Warsaw, Poland; mal2015lewandowska@gmail.com; 2 Division of Gynecological Surgery, University Hospital, 33 Polna Str., 60-535 Poznan, Poland

**Keywords:** macrosomia, prediction, obesity, weight gain, fetal sex, diabetes, pregnancy, factors

## Abstract

So far it has not been established which maternal features play the most important role in newborn macrosomia. The aim of this study is to provide assessment of a hierarchy of twenty six (26) maternal characteristics in macrosomia prediction. A Polish prospective cohort of women with singleton pregnancy (N = 912) which was recruited in the years 2015–2016 has been studied. Two analyses were performed: for probability of macrosomia > 4000 g (*n* = 97) (vs. 755 newborns 2500–4000 g); and for birthweight > 90th percentile (*n* = 99) (vs. 741 newborns 10–90th percentile). A multiple logistic regression was used (with 95% confidence intervals (CI)). A hierarchy of significance of potential predictors was established after summing up of three prediction indicators (NRI, IDI and AUC) calculated for the basic prediction model (maternal age + parity) extended with one (test) predictor. ‘Net reclassification improvement’ (NRI) focuses on the reclassification table describing the number of women in whom an upward or downward shift in the disease probability value occurred after a new factor had been added, including the results for healthy and ill women. ‘Integrated discrimination improvement’ (IDI) shows the difference between the value of mean change in predicted probability between the group of ill and healthy women when a new factor is added to the model. The area under curve (AUC) is a commonly used indicator. Results. The macrosomia risk was the highest for prior macrosomia (AOR = 7.53, 95%CI: 3.15–18.00, *p* < 0.001). A few maternal characteristics were associated with more than three times higher macrosomia odds ratios, e.g., maternal obesity and gestational age ≥ 38 weeks. A different hierarchy was shown by the prediction study. Compared to the basic prediction model (AUC = 0.564 (0.501–0.627), *p* = 0.04), AUC increased most when pre-pregnancy weight (kg) was added to the base model (AUC = 0.706 (0.649–0.764), *p* < 0.001). The values of IDI and NRI were also the highest for the model with maternal weight (IDI = 0.061 (0.039–0.083), *p* < 0.001), and (NRI = 0.538 (0.33–0.746), *p* < 0.001). Adding another factor to the base model was connected with significantly weaker prediction, e.g., for gestational age ≥ 38 weeks (AUC = 0.602 (0.543–0.662), *p* = 0.001), (IDI = 0.009 (0.004; 0.013), *p* < 0.001), and (NRI = 0.155 (0.073; 0.237), *p* < 0.001). After summing up the effects of NRI, IDI and AUC, the probability of macrosomia was most strongly improved (in order) by: pre-pregnancy weight, body mass index (BMI), excessive gestational weight gain (GWG) and BMI ≥ 25 kg/m^2^. Maternal height, prior macrosomia, fetal sex-son, and gestational diabetes mellitus (GDM) occupied an intermediate place in the hierarchy. The main conclusions: newer prediction indicators showed that (among 26 features) excessive pre-pregnancy weight/BMI and excessive GWG played a much more important role in macrosomia prediction than other maternal characteristics. These indicators more strongly highlighted the differences between predictors than the results of commonly used odds ratios.

## 1. Introduction

Numerous tests have revealed that newborns with excessive birth weight exhibit higher risk of birth complications and long-term negative health issues, such as diabetes and obesity [1,2,3,4]. Excessive birth weight can be an important element in the concept of noninfectious disease development according to the Developmental Origins of Health and Disease (DOHaD) and may result in a worse start to life compared to newborns with appropriate weight [2,5]. What is important, in well developed countries, is that the number of macrosomic newborns was reported to have increased from 5–20% to 15–25% [2,6]. Macrosomia is defined as birth weight above 4000 g regardless of gestational age; a large-for-gestational age (LGA) is defined as the weight > 90th percentile for the newborn sex and its gestational age, and for the particular population [1,7,8].

Early identification of macrosomia risk is necessary to provide the most effective early treatment. However, no standardized macrosomia treatment interventions have been determined yet [9]; this requires determination of the most important predictors. The literature confirms connection of maternal features with a higher macrosomia occurrence rate but the results of studies are diversified [1,2,3,10,11]. Maternal obesity and diabetes mellitus are considered to be independent factors of macrosomia risk, which has also been confirmed in meta-analyses [12,13]. However, women with appropriate weight and without diabetes mellitus also give birth to children with macrosomia [14]. At the same time the highest macrosomia odds ratio was reported for prior macrosomia [7]. The hierarchy of clinical factors in prediction of macrosomia is unknown, and the choice of its determination method is difficult.

Odds ratios (OR/AOR) of diseases have some limitations: e.g., incapability of comparing the impact of different continuous variables/risk factors (expressed in different units) or continuous variables with categorical variables; different results for different referential categories (in a study of dichotomous or categorical variables); decreasing the cohort size for a study of independent variables with missing data; different results after taking into consideration different cofounders.

A comparison of the significance of maternal characteristics in macrosomia risk is a challenge and should include many effects (mathematical and clinical). Newer probability indicators provide higher chances of finding out which of the predictors are more important [15]. Net Reclassification Improvement (NRI) calculates a few effects and assesses the percentage of persons (sick and healthy separately) in whom addition of the risk factor to the studied probability model improves or worsens the prediction (NRI provides a clinically favorable interpretation). Integrated Discrimination Improvement (IDI) calculates the mean change in disease probability after extension of the model with a new marker. In the analyses for NRI and IDI, missing data is a separate category and each analysis is based on the same data set. The value of another frequently used indicator, area under curve (AUC), also allows the comparison of different variables [15,16].

The goal of this study is to establish the significance hierarchy of 26 maternal characteristics as potential macrosomia predictors (in the literature referred to as macrosomia risk factors). Hierarchy of predictor significance was determined based on three prediction indicators (NRI, IDI and AUC) calculated after adding one (test) predictor to the base multifactorial prediction model. Adjusted odds ratios (AOR) of excessive birth weight were also calculated for each feature.

This is the first analysis of this type and is aimed at comparing the importance of different predictors, rather than duplicate the information that more risk factors increase the power of a prediction test.

## 2. Materials and Methods

The data used in this study came from a prospective cohort of Polish women with singleton pregnancy (N = 912). The recruitment included women at the end of the first trimester of pregnancy, and the mother and her pregnancy outcomes were recorded after the childbirth. The women did not suffer from any chronic diseases such as pre-existing diabetes or hypertension. This study was conducted in the Gynecology and Obstetrics University Hospital in Poznan, Poland (this is the third degree reference hospital for Obstetrics and Neonatology, with 6000–8000 births a year). The recruitment was conducted in the years 2015–2016.

### 2.1. Ethical Statement 

This study was consistent with the Helsinki Declaration. Participation in the study was voluntary and all participants had read and signed the Free Consent Form. The research process was accepted by the Bioethics Committee of the Medical University of Poznan, Poland (number 769/15).

### 2.2. Recruitment Criteria 

The inclusion criteria were as follows: women of Caucasian race from the Wielkopolski region, singleton pregnancy, 10–14th week of pregnancy (during recruitment), no fetus defects and 18–45 years old at the conception. Both primiparas and multiparas were subject to the study including their obstetric history (with or without macrosomic child births in previous pregnancy). Lack of chronic diseases in mothers apart from overweight and obesity (no diabetes mellitus, hypertension, immunological and inflammatory diseases, kidney and liver diseases, and thromboembolism) and use of mixed diet were also criteria for inclusion. 

The next recruitment criterion included a delivery of phenotypically normal child ≥ 25 weeks of pregnancy. The use of typical multivitamin/microelement preparations for pregnant women (including folic acid, vitamins B, C, D, E, A, as well as microelements such as iron, magnesium, calcium, selenium, copper, zinc, and manganese) was not among the criteria of exclusion/inclusion.

### 2.3. Method and Data Collection

Information about the research object and recruitment to the primary cohort was presented in the Central Laboratory (in the Clinical Hospital). The information was displayed in a place accessible for all pregnant women who were undergoing typical laboratory tests. 

In the first stage of the study, during recruitment of women in 10–14th week of pregnancy, 1300 women (meeting inclusion criteria) were willing to take part in the study (in the period of 12 months). After signing a conscious consent form all women completed a Survey Questionnaire about the course of pregnancy, obstetrical and gynecological histories, concurrent diseases, socio-economic and demographic characteristics, multivitamin preparations, medication, smoking of cigarettes, alcohol consumption, and record of family diseases. The women declared no alcohol during the pregnancy. The women completed the questionnaire on their own (in the presence of a midwife).

In the second stage of the study, after the childbirth, the information on maternal and neonatal outcomes was taken from medical documentation. Twelve-weeks after the childbirth, women informed the study coordinator (on the phone or by mail) about the course of pregnancy and the postnatal period, e.g., about smoking habit change or blood pressure profile during pregnancy or postnatal period (an additional questionnaire was completed).

After finishing the second stage of the study, 388 women were excluded from 1300: deliveries before 25th gestational week, cases of children with congenital diseases, cases of thromboembolism or severe infections during pregnancy, women with blood hypertension diagnosed before the 20th week and/or diabetes before the 18th week and lack of cooperation (*n* = 48), as well as women with significantly incomplete data sets (missing data) (*n* = 340). Finally, a cohort of 912 participants qualified for further analyses.

### 2.4. Minimum Sample Size

The study aimed to provide prediction of macrosomia in newborns for different maternal characteristics to find out which is the strongest predicator. The minimum sample size for this analysis was estimated by the formula for random recruitment:

n = Z^2^/d^2^ × p(1 − p) (1)

for the margin error d = 0.02 and confidence intervals 95%, α = 5% (“Z”—critical value of normal distribution at α/2, Z = 1.962), and the proportion of macrosomia *p* = 10% based on the literature covering studies of Polish [17] or foreign populations [1,2,3,9] (i.e., the highest mean value was accepted “p” found in the literature). The calculated minimum sample size (for *p* = 10%) was 864. 

### 2.5. Basic Characteristics of the Examined Population

In the studied cohort (N = 912), 97 women (10.6%) gave birth to newborns with macrosomia > 4000 g; the control group included 755 women (82.8%) who gave birth to newborns with weight between 2500 g and 4000 g. The remaining 60 women (6.6%) gave birth to children whose weight was <2500 g. In the analyzed cohort, 99 women (10.9%) gave birth to LGA newborns, and the control group included 741 women (81.2%) who gave birth to newborns with 10th a 90th percentile. The remaining 72 women (7.9%) gave birth to <10th percentile newborns. Other pregnancy outcomes (or complications) included: 146 mothers (16%) developed gestational diabetes mellitus (GDM) including 21 cases treated with insulin (GDM-2); 137 mothers (15%) developed pregnancy-induced hypertension including 24 cases of preeclampsia (PE); 65 mothers (7.1%) gave birth before 37th week; and 382 mothers (41.9%) had cesarian cut. 

### 2.6. Definition of the Studied Birth Outcome (Dependent Variables)

In this analysis, a childbirth of a newborn with macrosomia was the studied dependent variable. Birthweight was measured (in grams) immediately after a delivery using an automatic device. Macrosomia was defined as birth weight > 4000 g regardless of gestational age. Large-for-gestational age newborns (LGA) were defined as birth weight > 90th percentile and the percentiles were estimated for gestational age and fetal gender, based on Polish percentile grids [18]. Two analyses were performed: for women who gave birth to newborns with macrosomia > 4000 g vs. newborns 2500–4000 g; and for LGA birthweight vs. newborns 10–90th percentile. Gestational age was assessed using ultrasound examination (crown-rump length, CRL, was assessed between 10th and 13th (+6 days) week). 

Among other pregnancy outcomes, gestational diabetes mellitus (GDM) and pregnancy-induced hypertension were also noted (from medical records). To diagnose GDM, an oral glucose tolerance test (2-h test, fasting) for 75 g of glucose was performed in 24–28th week of gestation. Diabetes GDM-1 was defined as diabetes with dietary treatment. Diabetes GDM-2 was defined as diabetes with insulin treatment. A reference category was lack of GDM.

Pregnancy-induced hypertension (PIH) was defined as systolic and diastolic blood pressure ≥ 140/90 mmHg, developing de novo after the 20th week of pregnancy (obtained in at least two measurements 4 h apart, and measured with an oscillometric device in a sitting position). Preeclampsia was diagnosed when hypertension was accompanied by de novo development of organ disorder (renal and/or hepatic dysfunction and/or thrombocytopenia, cerebral and/or visual symptoms, or pulmonary edema).

### 2.7. Potential Predictors of Macrosomia (Independent Variables) 

In the current analysis, maternal characteristics were potential predictors identified in the literature as potential risk factors of macrosomia/LGA [1,2,3,8,10,11,19,20,21,22,23,24,25,26,27,28,29,30,31].

### 2.8. Statistical Analyses

The goal of this study is to establish the significance hierarchy of 26 maternal characteristics as potential macrosomia predictors. PQstat v1.8.0 software was used for the analyses (PQstat (manufacturer), Poznań, Poland). The *p*-value less than 0.05 was assumed as statistically significant. To achieve the aim of the study, two analyses were performed: (1) for women with macrosomic newborns (97 cases) compared to women with newborns 2500–4000 g (755 controls); (2) for women with LGA newborns (99 cases) compared to women with newborns 10–90th percentile (741 controls).

(1) First of all, independent variables (risk factors from Table 1) were described in the case and control groups using median and mean values (and standard deviation, SD) or by the number (and percentage). The normality of the data distribution for continuous independent variables (maternal age, pre-pregnancy weight, BMI, height, GWG) and newborn characteristics (gestational age and weight) was assessed with the Shapiro-Wilk test. These continuous variables were not normally distributed and the Mann-Whitney U test was used for comparisons of the variables between the case and control group. The Chi-squared test or Fisher exact test were used for comparisons of categorical variables. Statistical significance is given (*p*-value). 

(2) Secondly, crude odds ratios (OR, with 95% confidence intervals (CI)) of macrosomia for each risk factor were calculated in univariable logistic regression. Adjusted odds ratios (AOR) with 95%CI were obtained in multiple logistic regression after being adjusted to maternal age, parity and pre-pregnancy BMI. The statistical significance (*p*-value) of OR/AOR was determined by Wald’s test.

(3) Thirdly, a base predictive model was chosen (acquired from different sources knowing that ‘maternal age’ and ‘parity’ are important predictive factors to be used in newborn birth weight analyses).

(4) Next, we checked which variables (regardless of age and parity) have macrosomia predictive potential (in relation to normal birth weight). First of all, we determined variables with high AUC improvement indexes, and high NRI and IDI.

Each indicator was calculated for the basic prediction model (maternal age + parity) and for models extended with one (test) predictor. For each of the three indicators, 95% confidence intervals (CI) and their statistical significance (*p*-value) were calculated. For the AUC, NRI and IDI analyses, normally distributed test statistics were found.

(5) Initially, the significance hierarchy of potential predictors was established for each indicator separately (AUC or NRI, or IDI), assigning the first place to the predictor with the highest index and the last place to the predictor with the lowest index. 

(6) Finally, the sum of the sequences obtained from AUC, NRI and IDI was calculated and a new order of the variables was given, showing those maternal features which presented the greatest improvement in prediction (first in the sequences).

Net reclassification improvement (NRI) focuses on the reclassification table describing the number of participants in whom a downward or upward shift in the probability value occurred after a new variable had been added, including the results for sick and healthy participants. Integrated discrimination improvement (IDI) shows the difference between the mean change in the probability between the group of healthy and sick women when a new predicator is added to the model. AUC, and area under ROC curve (receiver operating characteristic curve), are commonly used probability indicators. Statistically significance and high values obtained for AUC, NRI and IDI for extended models prove good predictive ability of the factor added to the basic regression model [15,16]. 

In the study, the analyzed potential predictors were assessed as continuous variables and/or categorical variables (c) and/or dichotomous variables. The special category (‘unknown’) was created for ‘missing data’, which allowed us to evaluate all the predictors for the full set of participants.

Comments:

Our results showed that the general size of a cohort equal to 865 was good enough to discover interesting differences (statistically significant differences). 

All probability based predictors determined from the logistic regression model, including even the smallest probability increase or drop, exhibited high sensitivity not only to a change in the majority class but also in the minority class. 

Additionally, a prediction quality analysis for particular variables of a model including ‘maternal age’ and ‘parity’ gives an insight, unrelated to these two variables, into the contribution to high birth weight prediction. The base model is not big (only two variables); this enables assessment of many prognostic factors, because such a small model enables safe introduction of other variables and avoidance of redundancy.

## 3. Results

### 3.1. Basic Characteristics of the Women

Table S1 presents the basic characteristics of the participants. The whole studied cohort consisted of 912 women with a singleton pregnancy, who had no pre-existing diseases. The median of maternal age was 35 years (25–75% ranges 31–37), pre-pregnancy body mass index (BMI) 22.8 kg/m^2^ (25–75% ranges 20.6–26.1), and gestational weight gain (GWG) 13.8 kg (25–75% ranges 10–17). In the entire cohort there were 271 (29.7%) women with BMI ≥ 25 kg/m^2^, among them 98 (10.8%) obese women (BMI ≥ 30 kg/m^2^). The percentage of women with GWG above the Institute of Medicine (IOM, 2009) recommendation range was 36.8%. In the whole cohort, 97 women (10.6%) gave birth to newborns with macrosomia > 4000 g, and 60 (6.6%) had newborns < 2500 g (Table S1). 

Table 2 presents the characteristics of the women who gave birth to newborns with macrosomia > 4000 g.

The mothers of macrosomic newborn vs. mothers of newborn 2500–4000 g (Table 2) had higher mean values of height, BMI and GWG and more frequently reported prior macrosomia (13.4% vs. 1.7%), and these differences were statistically significant. Male fetal sex was also associated with cases of macrosomia (69.1% vs. 50.2%, *p* < 0.001). In the macrosomia group, there were also more cases of primiparous women, cases of gestational diabetes mellitus (GDM) including diabetes treated with insulin (GDM-2), as well as family history of diabetes mellitus (DM), but these differences were insignificant. The mean birth weight of children with macrosomia was 4245.6 g and was higher by 26.5% than average birth normal weight (3356.7 g).

### 3.2. Adjusted Odds Ratios of Macrosomia for Maternal Characteristics

Table S2 shows adjusted odds ratios of macrosomia and LGA newborns for maternal characteristics, calculated in multiple logistic regression after adjustment to maternal age, parity and pre-pregnancy BMI.

Among continuous variables, pre-pregnancy BMI, maternal height and GWG were associated with the highest adjusted odds ratios of macrosomia and LGA. An increase in pre-pregnancy BMI by 1 kg/m^2^ resulted in an increase in macrosomia risk by 13% (AOR = 1.13, 95%CI: 1.08–1.18, *p* < 0.001) and LGA risk by 11% (AOR = 1.11, 95%CI: 1.07–1.16, *p* < 0.001). The associations between maternal age (years) and macrosomia were weaker (Table S2).

Among the dichotomous variables (Table S2), macrosomia risk was the highest for prior macrosomia (AOR = 7.53, 95%CI: 3.15–18.00), *p* < 0.001). A few maternal characteristics were associated with more than two-three times higher macrosomia odds ratios: birth ≥ 38th gestational week (AOR = 3.87, 95%CI: 1.18–12.69, *p* = 0.025), pre-pregnancy BMI ≥ 30 kg/m^2^ (AOR = 3.39, 95%CI: 1.97–5.84, *p* < 0.001); BMI ≥ 25 kg/m^2^ (AOR = 2.84, 95%CI: 1.84–4.39, *p* < 0.001); maternal height > 160 cm (AOR = 2.78, 95%CI: 1.33–5.78, *p* = 0.006); male fetal sex (AOR = 2.36, 95%CI: 1.48–3.77, *p* < 0.001); GWG above the range (AOR = 2.26, 95%CI: 1.44–3.54; *p* < 0.001). 

Other maternal characteristics were poorly and statistically insignificantly related to macrosomia risk: diabetes mellitus developed in pregnancy; GDM (including GDM treated with diet and insulin); marital status-married; lower education level (<12 years); lower financial status; interpregnancy intervals; prior gestational diabetes (Table S2). The adjusted odds ratio of macrosomia for GDM was AOR = 1.13 (95%CI: 0.65–1.97, *p* = 0.665).

### 3.3. Macrosomia Prediction 

Table 3, Table 4 and Table 5 and Tables S3–S6 present the values of three indexes (AUC, IDI, and NRI) for macrosomia and LGA prediction after the extension of a basic multifactorial predictive model. Bright colors (green for macrosomia, and orange for LGA) were used to highlight the strongest results in the tables; the more intensive color the higher value of the indicator. Maternal features are listed according to the hierarchy, from the highest to the lowest values (Table 3, Table 4 and Table 5).

#### 3.3.1. Basic Predictive Model and Extended Predictive Models for Macrosomia Probability; Separate Analyses for AUC, IDI and NRI Indexes 

Table 3 presents values of the area under the receiver operating characteristic curve (AUC) (with 95% confidence intervals) in macrosomia prediction for 26 variables. The basic multifactorial predictive model (maternal age as a continuous variable + parity categories) was found to be good, and extension with (tested) one risk factor can show how a one (added) factor will improve the prediction of the extended model. AUC for macrosomia for the base model was AUC = 0.564, 95%CI: 0.501–0.627, *p* = 0.040).

AUC in macrosomia prediction increased most strongly when pre-pregnancy weight (kg) was added to the base model (AUC = 0.706, 95%CI: 0.649–0.764, *p* < 0.001). 

Lower AUC values were obtained for models extended with pre-pregnancy BMI (AUC = 0.671, 95%CI: 0.611–0.731, *p* < 0.001) or excessive GWG (AUC = 0.656, 95%CI: 0.597–0.716, *p* < 0.001). Next, lower AUC value were obtained for models extended with prior macrosomia (AUC = 0.611, 95%CI: 0.547–0.674, *p* < 0.001) or gestational age ≥ 38 weeks (AUC = 0.602, 95%CI: 0.543–0.662, *p* = 0.001) and maternal height > 160 cm (AUC = 0.602, 95%CI: 0.544–0.659, *p* = 0.001). 

Significantly lower AUC was obtained for the model extended with GDM developed in this pregnancy (AUC = 0.573, 95%CI: 0.511–0.636, *p* = 0.019).

The results for LGA prediction (orange color) were similar (Table S3).

Table 4 presents values of IDI (with 95% confidence intervals) in macrosomia prediction. IDI shows the difference between the value of the mean change in the predicted probability between the group of sick and healthy women when a new factor is added to the base model. The highest IDI was obtained for the model extended with pre-pregnancy weight (kg) (IDI = 0.061, 95%CI: 0.039–0.083, *p* < 0.001). 

Lower IDI values were obtained for models extended with prior macrosomia (IDI = 0.044, 95%CI: 0.017–0.07, *p* = 0.001) or pre-pregnancy BMI as a continuous variable (IDI = 0.041, 95%CI: 0.023–0.059, *p* < 0.001). Significantly lower IDI was obtained for model extended with fetal sex-son (IDI = 0.015, 95%CI: 0.007–0.023, *p* < 0.001), gestational age ≥ 38 weeks (IDI = 0.009, 95%CI: 0.004–0.013), *p* < 0.001) or maternal height > 160 cm (IDI = 0.008, 95%CI: 0.003–0.013, *p* = 0.004) as well as for GDM (IDI = 0.002, 95%CI: −0.001–0.006, *p* = 0.195). The results for LGA prediction (orange color) were similar (Table S4).

Table 5 presents values of NRI (with 95% confidence intervals) in macrosomia prediction. Net Reclassification Improvement (NRI) focuses on the reclassification table describing the number of women in whom an upward or downward shift in the disease probability value occurred after a new factor had been added (Table 5 and Table S5).

The highest NRI (Table 5) was obtained for the model with pre-pregnancy weight as a continuous variable (kg) (NRI = 0.538, 95%CI: 0.33–0.746, *p* < 0.001).

Lower NRI was obtained for models extended with: pre-pregnancy BMI categories (INR = 0.506, 95%CI: 0.298–0.715, *p* < 0.001); excessive GWG (NRI = 0.499, 95%CI: 0.293–0.706, *p* < 0.001); pre-pregnancy BMI ≥ 25 kg/m^2^ (NRI = 0.488, 95%CI: 0.280–0.697, *p* < 0.001).

Significantly lower NRI was obtained for models extended with maternal height > 160 cm (NRI = 0.214, 95%CI: 0.086–0.343, *p* = 0.001) or prior macrosomia (NRI = 0.159, 95%CI: −0.038–0.356, *p* = 0.114) as well as for GDM (NRI = 0.115, 95%CI: −0.057–0.287, *p* = 0.190).

The results for LGA prediction (orange color) were similar (Table S5).

NRI includes the results calculated for the ill group NRI(1), and for the healthy group NRI(0). The NRI(1) is an assessment for the sick participants, and it represents the difference in the value of probability increase and decrease in the model after adding a factor. In the analysis of macrosomia probability, for the model extended with pre-pregnancy weight (kg), NRI (1) = 13.4% means that in 13.4% of ill patients (women who gave birth to macrosomic newborns) there was a ‘correct’ reclassification in the direction of increased disease probability after adding pre-pregnancy weight (kg) to the model (56.7–43.3%) (Table 5). A detailed comment for macrosomia and LGA probability (NRI results) can be found under Table S5. 

#### 3.3.2. Graphical Pictures of Macrosomia and LGA Prediction (IDI, NRI and AUC Values)

**Figure 1:**
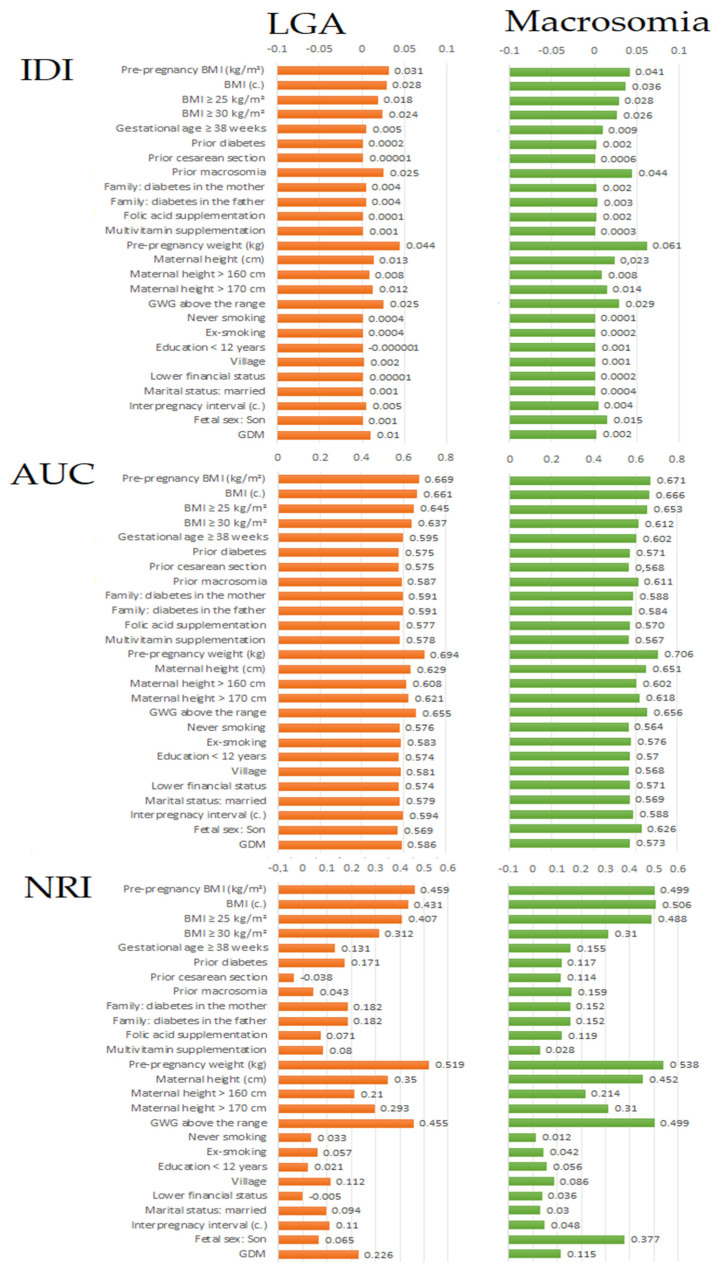
Values of each of the indices (IDI, AUC or NRI) in macrosomia and LGA prediction. The values were calculated after the extension of the basic multifactorial predictive model (maternal age + parity categories, 0, 1, 2, and ≥3 deliveries): one (test) variable was added to the base model. Macrosomia: birth weight > 4000 g (analysis for 97 cases vs. 755 newborns 2500–4000 g); LGA: birth weight > 90th percentile (analysis for 99 cases vs. 741 newborns 10–90th percentile); BMI: Body Mass Index; GWG: Gestational Weight Gain; GDM: gestational diabetes mellitus.

Figure 1 and Table S6 present basic values of the three indexes (AUC, IDI and NRI) in macrosomia prediction for 26 maternal characteristics.

It can be seen that prediction of both outcomes was most improved (the highest IDI, AUC and NRI indicators) after adding pre-pregnancy weight or pre pregnancy BMI to the base model.

Among the factors occupying an intermediate place in the hierarchy of predictors, all the prediction indicators, particularly NRI, showed that macrosomia prediction improved more significantly after adding (to the base model) male fetal sex rather than gestational diabetes mellitus (GDM); otherwise in LGA prediction. These two variables are presented at the bottom of the charts (Figure 1) and Table S6.

### 3.4. Final Order of Significance of Macrosomia and LGA Predictors; Overall Effects of Three Prediction Indexes (AUC, IDI and NRI)

**Figure 2:**
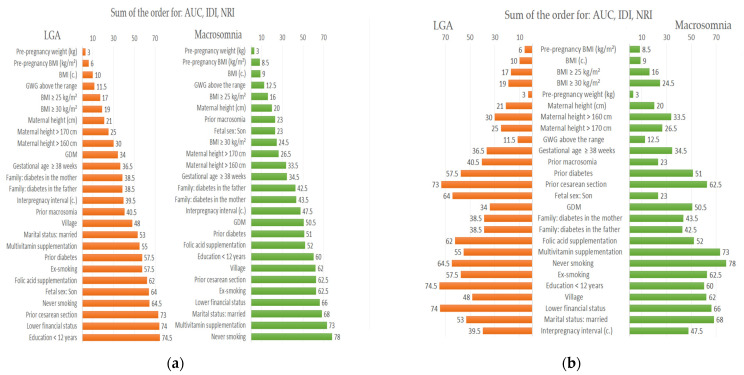
A new order of significance of the macrosomia and LGA predictors after the sum of the sequences obtained in the AUC, IDI and NRI analyses. The (**a**) image shows those clinical factors that most improved the prediction (ranking 1st in the sequences). The (**b**) image highlights differences between LGA and macrosomia for each predictor. Macrosomia: birth weight > 4000 g (analysis for 97 cases vs. 755 newborns 2500–4000 g); LGA: birth weight > 90th percentile (analysis for 99 cases vs. 741 newborns 10–90th percentile); BMI: Body Mass Index; GWG: Gestational Weight Gain; GDM: gestational diabetes mellitus; prior diabetes: i.e., prior GDM.

Figure 2 shows a new order of significance (ranking positions) of macrosomia and LGA predictors after summing up of the sequences obtained in the analyses of the three measures (AUC, IDI and NRI). The shorter the horizontal bar, the higher the position in the ranking of predictors. The (a) image shows those clinical factors that best improved predicting (ranking 1st in the sequences). The (b) image highlights differences between LGA and macrosomia for each predictor.

Figure 2 shows that after summing up the effects of three indexes (AUC, NRI, and IDI), the probability of both outcomes (macrosomia and LGA) was most strongly improved (in order) by: pre-pregnancy weight (kg) or BMI (kg/m^2^) as continuous variables; GWG above the range (the same results were obtained for GWG categories); BMI ≥ 25 kg/m^2^ as a dichotomous variable; and maternal height (cm) as a continuous variable.

The next predictors of macrosomia occupied middle positions in the hierarchy (in order): prior macrosomia, fetal sex-son, gestational age ≥ 38th week, family history of diabetes mellitus (DM), and gestational diabetes in current pregnancy (GDM). This was opposite to the LGA prediction where GDM and family history of diabetes mellitus (DM), and then prior macrosomia and fetal sex-son played a more important role.

At the bottom of macrosomia predictor and LGA were: prior cesarean section, prior diabetes (i.e., prior gestational diabetes mellitus), supplementing with multivitamins (typical preparations recommended to pregnant women (self-reported)), and socio-demographic-economic factors.

## 4. Discussion

In this assessment of the significance hierarchy of 26 maternal characteristics as potential macrosomia predictors, excessive pre-pregnancy weight/BMI and excessive GWG played the most important role in macrosomia prediction. This analysis based on newer prediction indices (NRI, IDI and AUC) showed that the importance of BMI and GWG was much higher than that of other maternal characteristics and this difference was stronger than the odds ratio results showed.

Although the role of maternal obesity and overweight in the development of excessive birth weight is known, the finding of such a strong role for these factors in macrosomia prediction confirms and emphasizes the need to improve the nutritional status of women before and during pregnancy in order to reduce the occurrence of this adverse newborn outcome. Additionally, the results of this study suggests that threshold BMI may be close to 25 kg/m^2^.

Comments on the applied statistical analyses can be found in Section 2.8.

The literature shows similar relationships between macrosomia and obesity/overweight (in studies of odds ratios) [1,9,13], but there are also discrepancies [7,42]. According to Akanmode et al., obesity is related to 4–12 times higher probability of macrosomia [1]. Other results show that the risk of macrosomia is 1.5–2.3 times higher in women with obesity as compared to women with proper BMI [9,13]. Excessive GWG also (though less so) increases macrosomia risk, which has also been confirmed in meta-analyses [32]. Nevertheless, Nkwabong et al. found statistically insignificant odds ratio of macrosomia for pre-pregnancy BMI ≥ 25 kg/m^2^ as compared to BMI < 25 (OR = 1.2 (95%CI 0.7–2.06)). In their study, GWG ≥ 16 kg as compared to GWG < 16 kg significantly increased macrosomia odds ratios (OR = 10.2 (95%CI: 4.5–22.9)), though newborns > 4000 g vs. newborns 3000–3999 g were examined [10]. Previous studies confirmed the relationship of macrosomia with other risk factors such as gestational diabetes mellitus (GDM), prior macrosomia, higher height of mother, fetus sex-son, family history of diabetes mellitus, and gestational age ≥ 38th week (the upper pregnancy age limit was 42 weeks) [1,2,3,10,14,39,43,44]. The role of other factors (prior cesarean section, prior gestational diabetes mellitus, multi-vitamin supplementation, and socio-demographic-economic factors) is also mentioned in the literature [20,22,30,39].

Inconsistencies between the results of the studies can be caused by a different methodology, including different sizes of the cohort, different control groups or different referential categories as well as different structures of the analyzed populations in terms of maternal age, BMI and obstetric history. According to this study, incidence of macrosomia (10.6%) was similar to the results provided by the literature [1,2,3,9,17]. In this study, 29.7% of the women had excessive pre pregnancy BMI ≥ 25 kg/m^2^ and most had excessive gestational weight gain (GWG) (59.0%), as compared to women with normal BMI (28.7%) (Table S1). The percentage of women with gestational diabetes mellitus GDM was 16%, including 2.3% for cases treated with insulin (GDM-2) and 13.7% for cases treated with dieting only (GDM-1), but this cohort had no chronic diseases (no preexisting diabetes mellitus). The participants in the current study came from one region, which matched the case and control groups in terms of the quality of prenatal care (including supplementation with multi-element multi-vitamin preparations), and the quality of diet in the region, and ruled out ethnic/racial factors.

In the current study, prior macrosomia and gestational diabetes mellitus (GDM) also occupied an intermediate place in the hierarchy of predictors; however, prior macrosomia was associated with the highest odds ratios of macrosomia (Table S2) and this is consistent with some literature reports [7,12]. In this study, GDM was associated with statistically insignificantly higher macrosomia odds ratio. This could be affected by the research methodology and lack of pre-existing diabetes, and low percentage of insulin treated GDM. GDM is a known macrosomia risk factor [1,12,45]. Meta-analysis by He et al. has shown that GDM statistically significantly increased macrosomia risk regardless of the impact of other factors (corrected odds ratio 1.71 (95%CI: 1.52–1.94)) [12]. 

In the current research, maternal age and parity are included in the base prediction model, therefore they were not considered to be ‘tested’ predictors. In a few studies, older maternal age and a bigger number of childbirths were also associated with higher macrosomia odds ratios [2,14,33].

The next finding was discovery/confirmation of a high convergence of the hierarchy of macrosomia and LGA predictors. Small differences were probably caused by the structure of the case and control groups.

The mechanisms linking maternal obesity/overweight (and excessive GWG) with higher risk of macrosomia in children are not fully explained. A newborn’s weight is affected by genetic factors, placental factors and mother’s nutrition [1,19,46,47]. It is believed that the underlying cause of macrosomia can be disorders accompanying obesity, such as insulin resistance and hyper insulinemia as well as dysregulation of functioning of numerous neurohormones and cytokines, chronic inflammation, intensive oxidative stress, and epigenetic changes [1,45,48]. Among others, intensified placental transport of glucose and amino acids to the fetus and its higher mass were found in women and animals using a diet that favors obesity [45,49,50,51]. Changed transport of lipids in the placenta was found in obese women with GDM which can contribute to fetus overgrowth [52]. In the literature, the need for effective control of glycaemia is also emphasized during pregnancy in women without GDM, as a factor which is connected with macrosomia risk [7,14]. 

Well prepared randomized intervention tests concerning diet and physical exercises are necessary to support women who plan pregnancy and who are pregnant. Multi center research performed by Poston et al. did not reveal any significant macrosomia incidence changes in obese pregnant women whose life style was changed during pregnancy [53], which can suggest the need to take an intervention in women before pregnancy [7]. This study may suggest the need to optimize to values less then BMI < 25 kg/m^2^. At the same time, randomized studies provided good effects in the area of dietetic counselling in women with GDM [3]. In our country, nutrition standards for pregnant women are similar to the recommendations of the World Health Organization (WHO) and the European Food Safety Authority (EFSA) or the Institute of Medicine (IOM) [54], as described in our earlier study [25]. The quality of diet is also important (also shown by our earlier studies) [46,54,55]. Recommendations of optimal weight gain in pregnancy (GWG) are adjusted to pre-pregnancy weight (BMI), according to the recommendations of the Institute of Medicine (IOM) in 2009 [14,21,32].

Obviously, the goal of this study does not cover all issues connected with macrosomia risk, i.e., the problems associated with ultrasound prenatal diagnostics, diagnostic thresholds, definitions, screening tests including biochemical markers, and different risk factors, as well as those involving prenatal care guidelines and delivery method [7,34]. These issues have been widely described [1,3,7,34]. 

### Limitations and Benefits

This study has a few advantages. It was performed for a prospectively collected cohort, i.e., during the recruitment the pregnancy results were not known. Prediction of macrosomia was evaluated separately > 4000 g and LGA. When determining the predictor significance hierarchy, assessment of a few prediction indicators was taken into consideration, including those indicators which evaluate reclassification on the basis of prediction improvement or worsening, including the effects both in the groups of sick and healthy patients. Independent variables (predicators) were assessed and expressed as continuous and/or categorical and/or dichotomous variables. 

This study has also some limitations. Although exclusion of chronic diseases such as pre-existing diabetes mellitus reduces excessive number of confounders, this probably contributed to a small share of effects such as development of cases of gestational diabetes mellitus treated with insulin (GDM-2) or prior GDM. A study of the role of physical exercise would be an interesting supplementation of the results. Pre-pregnancy weight was self-reported, which is a common practice. Comments on the statistical analyses used in the research can be found in Section 2.8.

## 5. Conclusions

In this study, among 26 analyzed risk factors, pre-pregnancy weight/BMI and gestational weight gain (GWG) were found to be the strongest predictors of macrosomia (>4000 g). The significance of other predictors was significantly lower. These results may suggest and highlight the need to reduce maternal weight (before and during pregnancy), in order to reduce the occurrence of macrosomia. However, well prepared randomized intervention studies are necessary to support women who plan pregnancy and who are pregnant.

## Figures and Tables

**Table 1:** Definitions of macrosomia risk factors identified from prior studies.

Risk Factor	Categories and Definitions	Reference Category	Description
Maternal age [25]	(as completed age at conception) - was assessed as a continuous variable (years) and - was assessed in the 5 following categories: (1) 18–24; (2) 25–29; (3) 30–34; (4) 35–39; (5) ≥40 years).	18–24 years; <40 years	From medical reports
Maternal height [24,26]	- was assessed as a continuous variable (cm) and - was assessed in the 4 following categories: (1) >170 cm; (2) ≤170 cm; (3) >160 cm; (4) ≤160 cm	≤170 cm; ≤160 cm	From medical reports
Pre-pregnancy weight [2,3,13,17]	was assessed as a continuous variable (kg)		Self-reported
Pre-pregnancy BMI [3,9,13,17]	(was calculated as the quotient of weight (in kg) and height (in meters) squared) - was assessed as a continuous variable (kg/m^2^) and - was assessed in the 4 following categories: (1) underweight (<18.5); (2) normal weight (18.5–24.9); (3) overweight (25.0–29.9); (4) obesity (≥30)	<25 kg/m^2^; <30 kg/m^2^	Self-reported
GWG [2,7,32]	(was defined as the difference between the weight before childbirth and the weight before pregnancy) - was assessed as a continuous variable (kg) and - was assessed in the 3 following categories (according to the 2009 Institute of Medicine recommendations): (1) above the range; (2) in the range; (3) below the range.	GWG other than above the range	From medical reports
GDM [3,12,21]	was assessed in the 4 following categories: (1) GDM; (2) diabetes with dietary treatment (GDM-1; (3) diabetes with insulin treatment (GDM-2); (4) no GDM.	No GDM	From medical reports
Parity [7,9,33]	was assessed in the five following categories: (1) primiparity i.e., zero delivery; (2) 1 delivery; (3) 2 deliveries; (4) ≥3 deliveries); (5) multiparity	Primiparity	From medical reports
Gestational age [7,29]	was assessed in the four following categories: (1) ≥38th week of gestation; (2) <38th week; (3) ≥40th week; (4) <40th week)	<38 weeks <40 weeks	From medical reports
Prior macrosomia [7,17]	was assessed in the two following categories: (1) prior macrosomia; (2) no prior macrosomia	No prior macrosomia	From medical reports
Prior GDM [7]	was assessed in the two following categories: (1) prior gestational diabetes mellitus, GDM; (2) no prior GDM	no prior GDM	From medical reports
Prior cesarean Section [4,34]	was assessed in the two following categories: (1) prior cesarean section; (2) no prior cesarean section	No prior cesarean section	From medical reports
Fetal sex [7,27]	was assessed in the two following categories: (1) son; (2) daughter	Daughter	From medical reports
Family -Diabetes mellitus in the mother [35,36]	was assessed in the three following categories: (1) diabetes in the mother; (2) diabetes in the family but not in the mother; (3) no diabetes in the family	No diabetes mellitus in the family	From medical reports
Family— Diabetes mellitus in the father [35,36]	was assessed in the three following categories: (1) diabetes in the father; (2) diabetes in the family but not in the father; (3) no diabetes in the family.	No diabetes mellitus in the family	From medical reports
Interpregnancy Interval [37]	was assessed in the seven following categories: (1) primigravida women; (2) 1 year; (3) 2 years; (4) 3–5 years; (5) 6–10 years; (6) ≥11 years; (7) the category “unknown” i.e., cases of missing data	1 year	From medical reports
Folic acid Supplementation [22]	was assessed in the two following categories: (1) supplementation with folic acid in the first trimester; (2) no folic acid supplementation in the first trimester	No folic acid supplementation	Self-reported and from medical reports
Multivitamin Supplementation [22]	was assessed in the two following categories: (1) supplementation with multi-vitamin-element preparations in the second-third trimester; (2) no multivitamin supplementation in the second-third trimester	No multivitamin supplementation	Self-reported and from medical reports
Never smokers [28]	was assessed in the three following categories: (1) never smokers; (2) ex-smokers (women who quit smoking before pregnancy); (3) smokers (before or during pregnancy)	Smokers	Self-reported
Education level [38]	was assessed in the three following categories: (1) <12 years of education (primary and vocational education); (2) ≥12 years of education (secondary education and tertiary education); (3) missing data	≥12 years of education	From medical reports
Financial status [39]	-was assessed on the 5-point Lickert scale. The basis of assessment was the question ‘is your household’s financial status sufficient for your needs?’ and the following answers: ‘1- definitely No’; ‘2- rather No’; ‘3- hard to say’; ‘4- rather Yes’; ‘5- definitely Yes’, - was assessed in the three following categories: (1) lower financial status (the answers 1-2-3); (2) higher financial status (the answers 4-5); (3) missing data	Higher financial status	Self-reported
Place of residence [40,41]	was assessed in the four following categories: (1) village; (2) small town < 50,000 residences; (3) large city > 50,000 residences; (4) missing data	Other than Village	From medical reports
Marital status [30]	was assessed in the four following categories: (1) married; (2) divorced; (3) unmarried; (4) missing data	Other than Married	From medical reports

BMI: body mass index; GWG: gestational weight gain; GDM: gestational diabetes mellitus.

**Table 2:** Basic characteristics of women who gave birth to newborns with macrosomia > 4000 g.

	Mothers of Newborn 2500–4000 g (*n* = 755)	Mothers of Newborn >4000 g (*n* = 97)	
Maternal Characteristics	Mean (SD)/Median or *n* (%)	Mean (SD)/Median or *n* (%)	p *
Continuous variables			
Maternal age (years)	33.5 (4.7)/35	34.4 (4.8)/35	0.120
Maternal height (cm)	166.4 (6.1)/167	169.2 (5.6)/170	<0.001
Pre-pregnancy BMI (kg/m^2^)	23.4 (4.2)/22	26.2 (5.4)/25	<0.001
GWG (kg)	13.5 (5.7)/13	15.7 (5.9)/15	0.001
Categorial and dichotomous variables			
BMI categories			0.001
Underweight (<18.5)	40 (5.3%)	4 (4.1%)	
Normal BMI (18.5–24.9)	518 (68.6%)	44 (45.4%)	
Overweight (25.0–29.9)	135 (17.9%)	26 (26.8%)	
Obesity (BMI ≥ 30)	62 (8.2%)	23 (23.7%)	
Prior fetal macrosomia	13 (1.7%)	13 (13.4%)	<0.001
Primiparous women	317 (42%)	38 (39.2%)	0.597
Infertility treatment	35 (4.6%)	4 (4.1%)	1 **
Never smokers	618 (81.9%)	80 (82.5%)	0.881
Multivitamins supplementation	441 (58.4%)	58 (59.8%)	0.795
Family history:			
Diabetes mellitus in the mother	53 (7%)	10 (10.3%)	0.244
Diabetes mellitus in the father	88 (11.7%)	16 (16.5%)	0.171
Pregnancy outcomes			
Birth weight (grams)	3356.7 (359)/3390	4245.6 (193.2)/4200	<0.001
(range, grams)	(2510–4000)	(4010–5200)	
Fetal sex/son	379 (50.2%)	67 (69.1%)	<0.001
Gestational age (weeks)	38.9 (1.3)/39	39.4 (1.1)/39	0.001
(range, weeks)	(30–42)	(37–42)	
Cesarean section	293 (38.8%)	46 (47.4%)	0.103
APGAR score 5′ < 7	0 (0%)	1 (1%)	0.114
GDM	119 (15.8%)	21 (21.6%)	0.141
GDM-2 (treated with insulin)	16 (2.1%)	3 (3.1%)	0.468
Preeclampsia, PE	10 (1.3%)	1 (1%)	1 **

* The Mann-Whitney U test was used for comparisons of continuous variables, and the Pearson chi-square test (or Fisher exact test when Cochran assumption was not met) for binomial categories (*p* < 0.05 was assumed to be significant); ** The result of the Fisher’s exact test was included due to the small numbers (additionally, the differences between the group of cases and controls were very small). SD: standard deviation; BMI: body mass index; GWG: gestational weight gain; APGAR: assessment of appearance, pulse, grimace, activity, and respiration; GDM: gestational diabetes mellitus.

**Table 3:** AUC values in the extended multivariate models for the probability of macrosomia.

	Macrosomia (>4000 g)					
Base Model (Maternal Age + Parity c **)	0.564	Base Model (0.501–0.627)	0.04	Differences *		
Extended Models (Base Model + Listed Variables)	AUC	±95%CI	*p*-Value	AUC Difference	±95%CI	*p* ***
Pre-pregnancy weight (kg)	0.706	(0.649–0.764)	<0.001	0.142	(0.077–0.208)	<0.001
Pre-pregnancy BMI (kg/m^2^)	0.671	(0.611–0.731)	<0.001	0.107	(0.045–0.17)	0.001
BMI (c.)	0.666	(0.607–0.724)	<0.001	0.102	(0.039–0.164)	0.001
GWG above the range	0.656	(0.597–0.716)	<0.001	0.092	(0.03–0.155)	0.004
BMI ≥ 25 kg/m^2^	0.653	(0.593–0.713)	<0.001	0.089	(0.029–0.149)	0.003
Maternal height (cm)	0.651	(0.595–0.707)	<0.001	0.087	(0.024–0.15)	0.007
Fetal sex: Son	0.626	(0.566–0.687)	<0.001	0.062	(0.007–0.118)	0.028
Maternal height > 170 cm	0.618	(0.556–0.679)	<0.001	0.054	(0–0.109)	0.055
BMI ≥ 30 kg/m^2^	0.612	(0.547–0.678)	<0.001	0.048	(0.007–0.089)	0.022
Prior macrosomia	0.611	(0.547–0.674)	<0.001	0.047	(0.009–0.084)	0.014
Gestational age ≥ 38 weeks	0.602	(0.543–0.662)	0.001	0.038	(0.01–0.066)	0.008
Maternal height > 160	0.602	(0.544–0.659)	0.001	0.038	(0–0.079)	0.077
Family: diabetes in the mother	0.588	(0.528–0.648)	0.005	0.024	(0–0.064)	0.238
Interpregnancy interval (c.)	0.588	(0.527–0.648)	0.005	0.024	(0–0.06)	0.2
Family: diabetes in the father	0.584	(0.523–0.644)	0.007	0.020	(0–0.057)	0.29
Ex–smoking	0.576	(0.515–0.637)	0.015	0.012	(0–0.034)	0.282
GDM	0.573	(0.511–0.636)	0.019	0.009	(0–0.039)	0.538
Prior diabetes	0.571	(0.509–0.634)	0.022	0.007	(0.002–0.012)	0.005
Lower financial status	0.571	(0.509–0.632)	0.023	0.007	(0–0.024)	0.439
Folic acid supplementation	0.570	(0.508–0.632)	0.025	0.006	(0–0.04)	0.724
Education < 12 years	0.57	(0.507–0.633)	0.024	0.006	(0–0.022)	0.432
Marital status: married	0.569	(0.507–0.631)	0.027	0.005	(0–0.024)	0.602
Prior cesarean section	0.568	(0.505–0.631)	0.029	0.004	(0–0.022)	0.666
Village	0.568	(0.504–0.633)	0.028	0.004	(0–0.029)	0.728
Multivitamin supplementation	0.567	(0.504–0.629)	0.032	0.003	(0–0.019)	0.728
Never smoking	0.564	(0.501–0.627)	0.04	0.000	(0–0.006)	0.998

AUC: area under receiver operating characteristic curve; 95%CI: confidence intervals; * Differences between extended models and base model; ** parity (c) categories: 0, 1, 2 and ≥3 deliveries); *** *p*-Value < 0.05 was statistically significant. Macrosomia: birth weight > 4000 g (analysis for 97 cases vs. 755 newborns 2500–4000 g); BMI: body mass index; GWG: gestational weight gain; GDM: gestational diabetes mellitus.

**Table 4:** Values of IDI in the extended multivariate models for the probability of macrosomia.

Extended Models (Base Model + Listed Variables) ***	IDI (95%CI) *	*p* **
	Macrosomia	
Pre-pregnancy weight (kg)	0.061(0.039; 0.083)	<0.001
Prior macrosomia	0.044(0.017; 0.07)	0.001
Pre-pregnancy BMI (kg/m^2^)	0.041(0.023; 0.059)	<0.001
BMI (c.)	0.036(0.02; 0.052)	<0.001
GWG above the range	0.029(0.017; 0.042)	<0.001
BMI ≥ 25 kg/m^2^	0.028(0.015; 0.041)	<0.001
BMI ≥ 30 kg/m^2^	0.026(0.011; 0.041)	0.001
Maternal height (cm)	0.023(0.012; 0.034)	<0.001
Fetal sex: Son	0.015(0.007; 0.023)	<0.001
Maternal height > 170 cm	0.014(0.005; 0.024)	0.003
Gestational age ≥ 38 weeks	0.009(0.004; 0.013)	<0.001
Maternal height > 160	0.008(0.003; 0.013)	0.004
Interpregnancy interval (c.)	0.004(−0.0005; 0.009)	0.080
Family: diabetes in the father	0.003(−0.001; 0.008)	0.138
Prior diabetes	0.002(0.0008; 0.003)	0.001
Family: diabetes in the mother	0.002(−0.001; 0.006)	0.205
Folic acid supplementation	0.002(−0.001; 0.004)	0.286
GDM	0.002(−0.001; 0.006)	0.195
Education < 12 years	0.001(−0.002; 0.003)	0.606
Village	0.001(−0.001; 0.003)	0.317
Prior cesarean section	0.0006(−0.001; 0.002)	0.515
Marital status: married	0.0004(−0.0009; 0.002)	0.544
Multivitamin supplementation	0.0003(−0.0009; 0.001)	0.655
Ex-smoking	0.0002(−0.001; 0.002)	0.834
Lower financial status	0.0002(−0.001; 0.001)	0.736
Never smoking	0.00009(−0.0003; 0.0004)	0.663

* IDI (95%CI): Integrated Discrimination Improvement (95% confidence intervals); ** *p*-Value < 0.05 was statistically significant; *** Base model: maternal age + parity categories (i.e., 0, 1, 2 and ≥3 deliveries). Macrosomia: birth weight > 4000 g (analysis for 97 cases vs. 755 newborns 2500–4000 g); BMI: body mass index; GWG: gestational weight gain; GDM: gestational diabetes mellitus.

**Table 5:** Values of NRI in the extended multivariate models for the probability of macrosomia.

Extended Models *** (Base Model + Listed Variables)	NRI (95%CI) *	*p* **	NRI(1)	NRI(0)	Ill = 1 Down|Up	Healthy = 0 Down|Up
	Macrosomia					
Pre-pregnancy weight (kg)	0.538(0.33; 0.746)	<0.001	13.4%	40.4%	43.3%|56.7%	70.2%|29.8%
BMI (c.)	0.506(0.298; 0.715)	<0.001	3.1%	47.5%	48.45%|51.55%	73.77%|26.23%
Pre-pregnancy BMI (kg/m^2^)	0.499(0.291; 0.708)	<0.001	9.3%	40.7%	45.36%|54.64%	70.33%|29.67%
GWG above the range	0.499(0.293; 0.706)	<0.001	19.6%	30.3%	40.21%|59.79%	65.17%|34.83%
BMI ≥ 25 kg/m^2^	0.488(0.28; 0.697)	<0.001	1.0%	47.8%	49.48%|50.52%	73.91%|26.09%
Maternal height (cm)	0.452(0.248; 0.656)	<0.001	27.8%	17.4%	36.08%|63.92%	58.68%|41.32%
Fetal sex: Son	0.377(0.18; 0.575)	<0.001	38.1%	-0.4%	30.93%|69.07%	49.8%|50.2%
BMI ≥ 30 kg/m^2^	0.31(0.136; 0.484)	<0.001	−52.6%	83.6%	76.29%|23.71%	91.79%|8.21%
Maternal height > 170 cm	0.31(0.109; 0.512)	0.002	−25.8%	56.8%	62.89%|37.11%	78.41%|21.59%
Maternal height > 160	0.214(0.086; 0.343)	0.001	81.4%	−60.0%	9.28%|90.72%	20%|80%
Prior macrosomia	0.159(−0.038; 0.356)	0.114	−34.0%	49.9%	67.01%|32.99%	74.97%|25.03%
Gestational age ≥ 38 weeks	0.155(0.073; 0.237)	<0.001	93.8%	−78.3%	3.09%|96.91%	10.86%|89.14%
Family: diabetes in the mother	0.152(−0.051; 0.355)	0.143	−25.8%	40.9%	62.89%|37.11%	70.46%|29.54%
Family: diabetes in the father	0.152(−0.051; 0.355)	0.143	−25.8%	40.9%	62.89%|37.11%	70.46%|29.54%
Folic acid supplementation	0.119(−0.09; 0.328)	0.264	−13.4%	25.3%	56.7%|43.3%	62.65%|37.35%
Prior diabetes	0.117(−0.055; 0.29)	0.183	58.8%	−47.0%	20.62%|79.38%	26.49%|73.51%
GDM	0.115(−0.057; 0.287)	0.190	−56.7%	68.2%	78.35%|21.65%	84.11%|15.89%
Prior cesarean section	0.114(−0.086; 0.313)	0.264	−32.0%	43.3%	65.98%|34.02%	71.66%|28.34%
Village	0.086(−0.111; 0.282)	0.393	−36.1%	44.6%	68.04%|31.96%	72.32%|27.68%
Education < 12 years	0.056(−0.065; 0.176)	0.365	−81.4%	87.0%	90.72%|9.28%	93.51%|6.49%
Interpregnancy interval (c.)	0.048(−0.158; 0.253)	0.649	23.7%	−18.9%	38.14%|61.86%	40.53%|59.47%
Ex-smoking	0.042(−0.105; 0.19)	0.574	−71.1%	75.4%	85.57%|14.43%	87.68%|12.32%
Lower financial status	0.036(−0.116; 0.188)	0.639	−69.1%	72.7%	84.54%|15.46%	86.36%|13.64%
Marital status: married	0.03(−0.157; 0.217)	0.752	−46.4%	49.4%	73.2%|26.8%	74.7%|25.3%
Multivitamin supplementation	0.028(−0.18; 0.235)	0.794	19.6%	−16.8%	40.21%|59.79%	41.59%|58.41%
Never smoking	0.012(−0.149; 0.173)	0.880	64.9%	−63.7%	17.53%|82.47%	18.15%|81.85%

* NRI (95%CI): Net Reclassification Improvement (95% confidence intervals); ** *p*-Value < 0.05 was statistically significant; *** Base model: maternal age + parity categories (i.e., 0, 1, 2 and ≥3 deliveries). Macrosomia: birth weight > 4000 g (analysis for 97 cases vs. 755 newborns 2500–4000 g); BMI: body mass index; GWG: gestational weight gain; GDM: gestational diabetes mellitus.

## Data Availability

The data presented in this study are available on request from the corresponding author. The data are not publicly available as it contains a variety of patient information and covers a much wider range than needed for the analyzes presented here.

## Supplementary Materials

The following are available online at https://www.mdpi.com/2072-6643/13/3/801/s1, Table S1: Basic characteristics of women with excessive pre-pregnancy BMI, Table S2: Odds ratios of macrosomia and LGA for selected maternal features, Table S3: Set of AUC values in the extended multivariate models for the probability of LGA and macrosomia, Table S4: Set of values of IDI in the extended multivariate models for the probability of LGA and macrosomia, Table S5: Set of values of NRI in the extended multivariate models for the probability of LGA and macrosomia, Table S6: Values of the three predictive indicators (AUC, IDI, NRI) in the extended multivariate models for the probability of LGA and macrosomia.

## Abbreviations

AOR	Adjusted odds ratio
AUC	Area under receiver operating characteristic curve (index)
BMI	Body mass index
CI	Confidence intervals
DM	Diabetes mellitus
DOHaD	Developmental Origins of Health and Disease
EFSA	European Food Safety Authority
GDM	Gestational diabetes mellitus
GWG	Gestational weight gain
IDI	Integrated Discrimination Improvement (index)
IOM	Institute of Medicine
LGA	Large-for-gestational age
NRI	Net Reclassification Improvement (index)
OR	Odds ratio
OGTT	Oral glucose tolerance test
PIH	Pregnancy-induced hypertension
PE	Preeclampsia
WHO	World Health Organization
