# Supplementary material for: The Role of Maternal Weight in the Hierarchy of Macrosomia Predictors; Overall Effect of Analysis of Three Prediction Indicators
Source: Nutrients. 2021 Feb 28;13(3):801. doi: 10.3390/nu13030801 (PMC8000437; doi:10.3390/nu13030801)
Supplement: Supplementary file 1 [file nutrients-13-00801-s001.zip › Table S1.docx]

**Table S1.** Basic characteristics of women with excessive pre-pregnancy BMI

|  | **Median (IQR) or n (%)** | **Median (IQR) or n (%)** | ***P* *** |
| --- | --- | --- | --- |
| **Characteristics** | **Normal**  **Pre-pregnancy BMI**  **(18.5−24.9 kg / m²)**  **(n = 593)** | **BMI ≥ 25 kg / m²**  **(n = 271)** |  |
| Continuous variable |  |  |  |
| Maternal age (years) | 35 (30-37) | 35 (32-37) | 0.003 |
| Pre-pregnancy weight (kg) | 60 (55-65) | 81 (75-89) | <0.0001 |
| Pre-pregnancy BMI (kg/m²) | 21.7 (20.3-23.2) | 28.7 (26.7-31.3) | <0.0001 |
| GWG (kg) | 14 (11-17) | 12 (8-17) | 0.0014 |
| BMI categories |  |  |  |
| Overweight | - | 173 |  |
| Obesity ≥ 30 kg / m² | - | 98 |  |
| Obesity I degree (BMI 30.0-34.9) | - | 70 |  |
| Obesity II degree (BMI 35-39.9) | - | 25 |  |
| Obesity III degree (BMI ≥ 40 kg/m²) | - | 4 |  |
| GWG categories |  |  | <0.0001 |
| GWG above the range | 170 (28.7%) | 160 (59%) |  |
| GWG in the range | 246 (41.5%) | 72 (26.6%) |  |
| GWG below the range | 177 (29.8%) | 39 (14.4%) |  |
| Primiparous women | 250 (42.2%) | 101 (37.3%) | 0.175 |
| Pregnancy outcomes |  |  |  |
| Birth weight (grams) |  |  | <0.0001 |
| <2500g | 32 (5.4%) | 25 (9.2%) |  |
| 2500−4000g | 517 (87.2%) | 197 (72.7%) |  |
| >4000g | 44 (7.4%) | 49 (18.1%) |  |
| PIH | 60 (10.1%) | 74 (27.3%) | <0.0001 |
| GDM | 79 (13.3%) | 58 (21.4%) | 0.003 |

* The Mann-Whitney U test was used for comparisons of continuous variables, For categorical ordered categories Cochran-Armitage test for trend was calculated, and for binomial categories the Pearson chi-square test (or Fisher exact test when Cochran assumption was not met) was used (*P* <0.05 was assumed to be significant). BMI: body mass index; GWG: gestational weight gain; PIH: pregnancy-induced hypertension GDM: gestational diabetes mellitus.
