# Supplementary material for: The Role of Maternal Weight in the Hierarchy of Macrosomia Predictors; Overall Effect of Analysis of Three Prediction Indicators
Source: Nutrients. 2021 Feb 28;13(3):801. doi: 10.3390/nu13030801 (PMC8000437; doi:10.3390/nu13030801)
Supplement: Supplementary file 1 [file nutrients-13-00801-s001.zip › Table S2.docx]

**Table S2.** Adjusted odds ratios of macrosomia and LGA for selected maternal features

|  | **Macrosomia risk** | **LGA risk** |
| --- | --- | --- |
| **Potential**  **predictors** | **AOR * (95% CI); *p*** | **AOR * (95% CI); *p*** |
| Continuous variables |  |  |
| Pre−pregnancy BMI (kg/m²) | **1.13** (1.08−1.18); <0.001 | **1.11** (1.07−1.16); <0.001 |
| Maternal height (cm) | **1.09** (1.05−1.13); <0.001 | **1.07** (1.03−1.11); 0.001 |
| GWG (kg) | **1.08** (1.04−1.12); <0.001 | **1.08** (1.04−1.12); <0.001 |
| Pre−pregnancy weight (kg) | **1.05** (1.04−1.07); <0.001 | **1.04** (1.03−1.06); <0.001 |
| Maternal age (years) | 1.01 (0.96−1.07); 0.616 | 1.02 (0.97−1.08); 0.426 |
| Dichotomous variables |  |  |
| Prior macrosomia (vs others) | **7.53** (3.15−18.00); <0.001 | **4.51** (1.89−10.74); 0.001 |
| Birth ≥ 38th week (vs < 38) | **3.87** (1.18−12.69); 0.025 | **2.40** (1.06−5.43); 0.036 |
| BMI ≥ 30 kg/m² (vs others) | **3.39** (1.97−5.84); <0.001 | **3.30** (1.94−5.63); <0.001 |
| BMI ≥ 25 kg/m² (vs others) | **2.84** (1.84−4.39); <0.001 | **2.34** (1.52−3.60); <0.001 |
| Height > 160 cm (vs others) | **2.78** (1.33−5.78); 0.006 | **2.63** (1.27−5.46); 0.009 |
| Height > 170 cm (vs others) | **2.32** (1.46−3.68); <0.001 | **2.16** (1.37−3.42); 0.001 |
| Fetal sex- son (vs others) | **2.36 (**1.48−3.77); <0.001 | 1.18 (0.77−1.82); 0.453 |
| GWG above the range (vs others) | **2.26** (1.44−3.54); <0.001 | **2.15** (1.38−3.35); 0.001 |
| Maternal age ≥ 40 years |  |  |
| (vs. < 40 years) | 1.89 (0.96−3.72); 0.064 | 1.48 (0.75−2.95); 0.262 |
| (vs. 18-24 years) ** | 8.95 (1.02−78.4); 0.048 | 4.53 (0.51−40.0); 0.174 |
| Parity; n ≥3 (vs n = 0) | 1.55 (0.56−4.26); 0.396 | 1.95 (0.75−5.1); 0.172 |
| Family history *** : |  |  |
| Diabetes in the father (vs Ref.) | 1.41 (0.76−2.62); 0.274 | 1.47 (0.79−2.72); 0.221 |
| Diabetes in the mother (vs Ref.) | 1.21 (0.57−2.57); 0.618 | 1.51 (0.73−3.12); 0.268 |
| Never smokers (vs others) | 1.26 (0.71−2.26); 0.431 | 1.24 (0.69−2.23); 0.473 |
| Ex−smokers (vs others) | 1.11 (0.59−2.07); 0.748 | 1.23 (0.67−2.25); 0.511 |
| Village (vs others) | 1.26 (0.79−2.01); 0.335 | 1.27 (0.81−2.0); 0.305 |
| Prior cesarean section (vs others) | 1.19 (0.66−2.14); 0.560 | 0.93 (0.53−1.66); 0.813 |
| GDM (vs no GDM) | 1.13 (0.65−1.97); 0.665 | 1.60 (0.95−2.70); 0.079 |
| Folic acid suppl. (vs others) | 1.16 (0.74−1.8); 0.516 | 1.08 (0.69−1.67); 0.748 |
| Multivitamins suppl. (vs others) | 1.09 (0.70−1.70); 0.699 | 1.26 (0.81−1.96); 0.303 |

* AOR: adjusted odds ratios (and 95% confidence intervals, CI) calculated in the multiple logistic regression after adjusted for maternal age, parity and pre-pregnancy BMI (*p*-value < 0.05 was statistically significant); ** The AOR was obtained after adjusted for many confounders**;** *** Reference category: no family history of diabetes mellitus; BMI: body mass index; GWG: gestational weight gain; GDM: gestational diabetes mellitus. Macrosomia: birth weight > 4000 g (analysis for 97 cases vs. 755 newborns 2500−4000 g); LGA: birth weight > 90th percentile (analysis for 99 cases vs. 741 newborns 10−90th percentile).
