# Supplementary material for: The Role of Maternal Weight in the Hierarchy of Macrosomia Predictors; Overall Effect of Analysis of Three Prediction Indicators
Source: Nutrients. 2021 Feb 28;13(3):801. doi: 10.3390/nu13030801 (PMC8000437; doi:10.3390/nu13030801)
Supplement: Supplementary file 1 [file nutrients-13-00801-s001.zip › Table S3.docx]

**Table S3.** Set of AUC values in the extended multivariate models for the probability of LGA and macrosomia

|  | **LGA ( > 90th percentile)** | | | |  |  |
| --- | --- | --- | --- | --- | --- | --- |
| *Base model*  *(maternal age + parity c **)* | *0.574* | *Base model*  *(0.513-0.635)* | *0.017* | *Differences ** |  |  |
| **Extended models**  **(base model + listed variables)** | **AUC** | **± 95% CI** | **p **** | **AUC difference** | **± 95% CI** | ***p* ***** |
| Pre-pregnancy weight (kg) | 0.694 | (0.638−0.751) | <0.001 | 0.120 | (0.055−0.186) | <0.001 |
| Pre−pregnancy BMI (kg/m²) | 0.669 | (0.611−0.726) | <0.001 | 0.095 | (0.034−0.156) | 0.002 |
| BMI (c.) | 0.661 | (0.602−0.72) | <0.001 | 0.087 | (0.028−0.146) | 0.004 |
| GWG above the range | 0.655 | (0.597−0.713) | <0.001 | 0.081 | (0.02−0.142) | 0.009 |
| BMI ≥ 25 kg/m² | 0.645 | (0.588−0.703) | <0.001 | 0.071 | (0.016−0.127) | 0.011 |
| BMI ≥ 30 kg/m² | 0.637 | (0.575−0.699) | <0.001 | 0.063 | (0.018−0.109) | 0.006 |
| Maternal height (cm) | 0.629 | (0.573−0.685) | <0.001 | 0.055 | (0.001−0.109) | 0.045 |
| Maternal height > 170 cm | 0.621 | (0.561−0.681) | <0.001 | 0.047 | (0−0.099) | 0.075 |
| Maternal height > 160 | 0.608 | (0.551−0.665) | <0.001 | 0.034 | (0−0.07) | 0.061 |
| Gestational age ≥ 38 weeks | 0.595 | (0.536−0.654) | 0.002 | 0.021 | (0−0.059) | 0.262 |
| Interpregnancy interval (c.) | 0.594 | (0.533−0.656) | 0.002 | 0.02 | (0−0.057) | 0.27 |
| Family: diabetes in the mother | 0.591 | (0.531−0.651) | 0.003 | 0.017 | (0−0.055) | 0.367 |
| Family: diabetes in the father | 0.591 | (0.531−0.651) | 0.003 | 0.017 | (0−0.055) | 0.374 |
| Prior macrosomia | 0.587 | (0.523−0.65) | 0.005 | 0.013 | (0−0.032) | 0.198 |
| GDM | 0.586 | (0.524−0.648) | 0.005 | 0.012 | (0−0.052) | 0.544 |
| Ex−smoking | 0.583 | (0.525−0.642) | 0.007 | 0.009 | (0−0.032) | 0.389 |
| Village | 0.581 | (0.519−0.644) | 0.008 | 0.007 | (0−0.034) | 0.572 |
| Marital status: married | 0.579 | (0.518−0.64) | 0.011 | 0.005 | (0−0.024) | 0.599 |
| Multivitamin supplementation | 0.578 | (0.518−0.638) | 0.012 | 0.004 | (0−0.031) | 0.749 |
| Folic acid supplementation | 0.577 | (0.518−0.635) | 0.013 | 0.003 | (0−0.021) | 0.756 |
| Never smoking | 0.576 | (0.514−0.639) | 0.013 | 0.002 | (0−0.011) | 0.499 |
| Prior diabetes | 0.575 | (0.514−0.636) | 0.015 | 0.001 | (0−0.014) | 0.853 |
| Prior cesarean section | 0.575 | (0.513−0.636) | 0.016 | 0.001 | (0−0.003) | 0.514 |
| Education < 12 years | 0.574 | (0.513−0.635) | 0.017 | 0 | (0−0.002) | 0.896 |
| Lower financial status | 0.574 | (0.513−0.635) | 0.017 | 0 | (0−0.001) | 0.756 |
| Fetal sex: Son | 0.569 | (0.509−0.629) | 0.026 | −0.005 | (0−0.021) | 0.563 |
|  | **Macrosomia ( > 4000 g)** | | | |  |  |
| *Base model*  *(maternal age + parity c **)* | 0.564 | *Base model*  *(0.501−0.627)* | *0.04* | *Differences ** |  |  |
| **Extended models**  **(base model + listed variables)** | **AUC** | **± 95% CI** | **p−value** | **AUC difference** | **± 95% CI** | ***p* ***** |
| Pre−pregnancy weight (kg) | 0.706 | (0.649−0.764) | <0.001 | 0.142 | (0.077−0.208) | <0.001 |
| Pre−pregnancy BMI (kg/m²) | 0.671 | (0.611−0.731) | <0.001 | 0.107 | (0.045−0.17) | 0.001 |
| BMI (c.) | 0.666 | (0.607−0.724) | <0.001 | 0.102 | (0.039−0.164) | 0.001 |
| GWG above the range | 0.656 | (0.597−0.716) | <0.001 | 0.092 | (0.03−0.155) | 0.004 |
| BMI ≥ 25 kg/m² | 0.653 | (0.593−0.713) | <0.001 | 0.089 | (0.029−0.149) | 0.003 |
| Maternal height (cm) | 0.651 | (0.595−0.707) | <0.001 | 0.087 | (0.024−0.15) | 0.007 |
| Fetal sex: Son | 0.626 | (0.566−0.687) | <0.001 | 0.062 | (0.007−0.118) | 0.028 |
| Maternal height > 170 cm | 0.618 | (0.556−0.679) | <0.001 | 0.054 | (0−0.109) | 0.055 |
| BMI ≥ 30 kg/m² | 0.612 | (0.547−0.678) | <0.001 | 0.048 | (0.007−0.089) | 0.022 |
| Prior macrosomia | 0.611 | (0.547−0.674) | <0.001 | 0.047 | (0.009−0.084) | 0.014 |
| Gestational age ≥ 38 weeks | 0.602 | (0.543−0.662) | 0.001 | 0.038 | (0.01−0.066) | 0.008 |
| Maternal height > 160 | 0.602 | (0.544−0.659) | 0.001 | 0.038 | (0−0.079) | 0.077 |
| Family: diabetes in the mother | 0.588 | (0.528−0.648) | 0.005 | 0.024 | (0−0.064) | 0.238 |
| Interpregnancy interval (c.) | 0.588 | (0.527−0.648) | 0.005 | 0.024 | (0−0.06) | 0.2 |
| Family: diabetes in the father | 0.584 | (0.523−0.644) | 0.007 | 0.020 | (0−0.057) | 0.29 |
| Ex−smoking | 0.576 | (0.515−0.637) | 0.015 | 0.012 | (0−0.034) | 0.282 |
| GDM | 0.573 | (0.511−0.636) | 0.019 | 0.009 | (0−0.039) | 0.538 |
| Prior diabetes | 0.571 | (0.509−0.634) | 0.022 | 0.007 | (0.002−0.012) | 0.005 |
| Lower financial status | 0.571 | (0.509−0.632) | 0.023 | 0.007 | (0−0.024) | 0.439 |
| Folic acid supplementation | 0.570 | (0.508−0.632) | 0.025 | 0.006 | (0−0.04) | 0.724 |
| Education < 12 years | 0.57 | (0.507−0.633) | 0.024 | 0.006 | (0−0.022) | 0.432 |
| Marital status: married | 0.569 | (0.507−0.631) | 0.027 | 0.005 | (0−0.024) | 0.602 |
| Prior cesarean section | 0.568 | (0.505−0.631) | 0.029 | 0.004 | (0−0.022) | 0.666 |
| Village | 0.568 | (0.504−0.633) | 0.028 | 0.004 | (0−0.029) | 0.728 |
| Multivitamin supplementation | 0.567 | (0.504−0.629) | 0.032 | 0.003 | (0−0.019) | 0.728 |
| Never smoking | 0.564 | (0.501−0.627) | 0.04 | 0.000 | (0−0.006) | 0.998 |

AUC: area under receiver operating characteristic curve; 95% CI: confidence intervals; * Differences between extended models and base model; ** parity (c) categories: 0, 1, 2 and ≥3 deliveries); *** *p*-Value <0.05 was statistically significant.

LGA: birth weight > 90th percentile (analysis for 99 cases vs. 741 newborns 10−90th percentile); Macrosomia: birth weight > 4000 g (analysis for 97 cases vs. 755 newborns 2500−4000 g); BMI: body mass index; GWG: gestational weight gain; GDM: gestational diabetes mellitus.
