# Supplementary material for: The Role of Maternal Weight in the Hierarchy of Macrosomia Predictors; Overall Effect of Analysis of Three Prediction Indicators
Source: Nutrients. 2021 Feb 28;13(3):801. doi: 10.3390/nu13030801 (PMC8000437; doi:10.3390/nu13030801)
Supplement: Supplementary file 1 [file nutrients-13-00801-s001.zip › Table S4.docx]

**Table S4.** Set of values of IDI in the extended multivariate models for the probability of LGA and macrosomia

| **Extended models**  **(base model + listed variables) ***** | **IDI (95% CI) *** | ***p* **** |
| --- | --- | --- |
|  | **LGA** |  |
| Pre-pregnancy weight (kg) | 0.044(0.025; 0.063) | <0.001 |
| Pre-pregnancy BMI (kg/m²) | 0.031(0.015; 0.047) | <0.001 |
| BMI (c.) | 0.028(0.013; 0.043) | <0.001 |
| Prior macrosomia | 0.025(0.007; 0.044) | 0.008 |
| GWG above the range | 0.025(0.013; 0.036) | <0.001 |
| BMI ≥ 30 kg/m² | 0.024(0.01; 0.039) | 0.001 |
| BMI ≥ 25 kg/m² | 0.018(0.007; 0.028) | 0.001 |
| Maternal height (cm) | 0.013(0.005; 0.021) | 0.002 |
| Maternal height > 170 cm | 0.012(0.004; 0.021) | 0.006 |
| GDM | 0.01(0.002; 0.017) | 0.016 |
| Maternal height > 160 | 0.008(0.003; 0.013) | 0.002 |
| Gestational age ≥ 38 weeks | 0.005(-0.0007; 0.01) | 0.024 |
| Interpregnancy interval (c.) | 0.005(-0.0002 ;0.011) | 0.061 |
| Family: diabetes in the mother | 0.004(-0.0002; 0.01) | 0.107 |
| Family: diabetes in the father | 0.004(-0.0002; 0.009) | 0.104 |
| Village | 0.002(-0.001; 0.004) | 0.240 |
| Multivitamin supplementation | 0.001(-0.001; 0.004) | 0.280 |
| Marital status: married | 0.0006(-0.001; 0.002) | 0.526 |
| Fetal sex: Son | 0.0005(-0.0009; 0.002) | 0.494 |
| Never smoking | 0.0004(-0.0004; 0.001) | 0.365 |
| Ex-smoking | 0.0004(-0.002; 0.003) | 0.696 |
| Prior diabetes | 0.0002(-0.0008; 0.001) | 0.636 |
| Folic acid supplementation | 0.0001(-0.002; 0.002) | 0.877 |
| Prior cesarean section | 0.00001(-0.0003; 0.00002) | 0.917 |
| Lower financial status | 0.000009(-0.00002; 0.00003) | 0.496 |
| Education < 12 years | -0.000001(-0.0002; 0.0002) | 0.986 |
|  | **Macrosomia** |  |
| Pre-pregnancy weight (kg) | 0.061(0.039; 0.083) | <0.001 |
| Prior macrosomia | 0.044(0.017; 0.07) | 0.001 |
| Pre-pregnancy BMI (kg/m²) | 0.041(0.023; 0.059) | <0.001 |
| BMI (c.) | 0.036(0.02; 0.052) | <0.001 |
| GWG above the range | 0.029(0.017; 0.042) | <0.001 |
| BMI ≥ 25 kg/m² | 0.028(0.015; 0.041) | <0.001 |
| BMI ≥ 30 kg/m² | 0.026(0.011; 0.041) | 0.001 |
| Maternal height (cm) | 0.023(0.012; 0.034) | <0.001 |
| Fetal sex: Son | 0.015(0.007; 0.023) | <0.001 |
| Maternal height > 170 cm | 0.014(0.005; 0.024) | 0.003 |
| Gestational age ≥ 38 weeks | 0.009(0.004; 0.013) | <0.001 |
| Maternal height > 160 | 0.008(0.003; 0.013) | 0.004 |
| Interpregnancy interval (c.) | 0.004(-0.0005; 0.009) | 0.080 |
| Family: diabetes in the father | 0.003(-0.001; 0.008) | 0.138 |
| Prior diabetes | 0.002(0.0008; 0.003) | 0.001 |
| Family: diabetes in the mother | 0.002(-0.001; 0.006) | 0.205 |
| Folic acid supplementation | 0.002(-0.001; 0.004) | 0.286 |
| GDM | 0.002(-0.001; 0.006) | 0.195 |
| Education < 12 years | 0.001(-0.002; 0.003) | 0.606 |
| Village | 0.001(-0.001; 0.003) | 0.317 |
| Prior cesarean section | 0.0006(-0.001; 0.002) | 0.515 |
| Marital status: married | 0.0004(-0.0009; 0.002) | 0.544 |
| Multivitamin supplementation | 0.0003(-0.0009; 0.001) | 0.655 |
| Ex-smoking | 0.0002(-0.001; 0.002) | 0.834 |
| Lower financial status | 0.0002(-0.001; 0.001) | 0.736 |
| Never smoking | 0.00009(-0.0003; 0.0004) | 0.663 |

* IDI (95%CI): Integrated Discrimination Improvement (95% confidence intervals); ** *p*-Value <0.05 was statistically significant; *** Base model: maternal age + parity categories (i.e. 0, 1, 2 and ≥3 deliveries). LGA: birth weight > 90th percentile (analysis for 99 cases vs. 741 newborns 10−90th percentile); Macrosomia: birth weight > 4000 g (analysis for 97 cases vs. 755 newborns 2500−4000 g); BMI: body mass index; GWG: gestational weight gain; GDM: gestational diabetes mellitus.
