# Supplementary material for: The Role of Maternal Weight in the Hierarchy of Macrosomia Predictors; Overall Effect of Analysis of Three Prediction Indicators
Source: Nutrients. 2021 Feb 28;13(3):801. doi: 10.3390/nu13030801 (PMC8000437; doi:10.3390/nu13030801)
Supplement: Supplementary file 1 [file nutrients-13-00801-s001.zip › Table S5.docx]

**Table S5.** Set of values of NRI in the extended multivariate models for the probability of LGA and macrosomia

| **Extended models**  **(base model + listed variables) ***** | **NRI (95% CI) *** | ***p* **** | **NRI(1)** | **NRI(0)** | **ill = 1**  **down \| up** | **healthy = 0**  **down \| up** |
| --- | --- | --- | --- | --- | --- | --- |
|  | **LGA** |  |  |  |  |  |
| Pre-pregnancy weight (kg) | 0.519(0.312; 0.725) | <0.001 | 13.1% | 38.7% | 43.43% \| 56.57% | 69.37% \| 30.63% |
| Pre-pregnancy BMI (kg/m²) | 0.459(0.252; 0.667) | <0.001 | 5.1% | 40.9% | 47.47% \| 52.53% | 70.45% \| 29.55% |
| GWG above the range | 0.455(0.249; 0.662) | <0.001 | 15.2% | 30.4% | 42.42% \| 57.58% | 65.18% \| 34.82% |
| BMI (c.) | 0.431(0.224; 0.638) | <0.001 | -1.0% | 44.1% | 50.51% \| 49.49% | 72.06% \| 27.94% |
| BMI ≥ 25 kg/m² | 0.407(0.2; 0.614) | <0.001 | -5.1% | 45.7% | 52.53% \| 47.47% | 72.87% \| 27.13% |
| Maternal height (cm) | 0.35(0.145; 0.554) | 0.001 | 23.2% | 11.7% | 38.38% \| 61.62% | 55.87% \| 44.13% |
| BMI ≥ 30 kg/m² | 0.312(0.138; 0.486) | <0.001 | -51.5% | 82.7% | 52.53% \| 47.47% | 72.87% \| 27.13% |
| Maternal height > 170 cm | 0.293(0.094; 0.491) | 0.004 | -27.3% | 56.5% | 63.64% \| 36.36% | 78.27% \| 21.73% |
| GDM | 0.226(0.045; 0.406) | 0.014 | -47.5% | 70.0% | 73.74% \| 26.26% | 85.02% \| 14.98% |
| Maternal height > 160 | 0.21(0.083; 0.336) | 0.001 | 81.8% | -60.9% | 9.09% \| 90.91% | 19.57% \| 80.43% |
| Family: diabetes in the mother | 0.182(; 0.021; 0.384) | 0.078 | -23.2% | 41.4% | 61.62% \| 38.38% | 70.72% \| 29.28% |
| Family: diabetes in the father | 0.182(; 0.021; 0.384) | 0.078 | -23.2% | 41.4% | 61.62% \| 38.38% | 70.72% \| 29.28% |
| Prior diabetes | 0.171(0.006; 0.336) | 0.042 | 63.6% | -46.6% | 18.18% \| 81.82% | 26.72% \| 73.28% |
| Gestational age ≥ 38 weeks | 0.131(0.019; 0.244) | 0.022 | 85.9% | -72.7% | 7.07% \| 92.93% | 13.63% \| 86.37% |
| Village | 0.112(-0.086; 0.31) | 0.268 | -31.3% | 42.5% | 65.66% \| 34.34% | 71.26% \| 28.74% |
| Interpregnancy interval (c.) | 0.11(-0.099; 0.318) | 0.302 | 11.1% | -0.1% | 44.44% \| 55.56% | 49.93% \| 50.07% |
| Marital status: married | 0.094(-0.079; 0.267) | 0.287 | 57.6% | -48.2% | 21.21% \| 78.79% | 25.91% \| 74.09% |
| Multivitamin supplementation | 0.08(-0.125; 0.284) | 0.444 | 23.2% | -15.2% | 38.38% \| 61.62% | 42.38% \| 57.62% |
| Folic acid supplementation | 0.071(-0.134; 0.277) | 0.497 | -19.2% | 26.3% | 59.6% \| 40.4% | 63.16% \| 36.84% |
| Fetal sex: Son | 0.065(-0.144; 0.274) | 0.540 | 9.1% | -2.6% | 45.45% \| 54.55% | 48.72% \| 51.28% |
| Ex-smoking | 0.057(-0.092; 0.206) | 0.450 | -69.7% | 75.4% | 84.85% \| 15.15% | 87.72% \| 12.28% |
| Prior macrosomia | 0.043(-0.143; 0.23) | 0.648 | -45.5% | 49.8% | 72.73% \| 27.27% | 74.9% \| 25.1% |
| Never smoking | 0.033(-0.122; 0.188) | 0.676 | 67.7% | -64.4% | 16.16% \| 83.84% | 17.81% \| 82.19% |
| Education < 12 years | 0.021(-0.092; 0.135) | 0.713 | -83.8% | 86.0% | 91.92% \| 8.08% | 92.98% \| 7.02% |
| Lower financial status | -0.005(-0.151; 0.141) | 0.948 | 71.7% | -72.2% | 14.14% \| 85.86% | 13.9% \| 86.1% |
| Prior cesarean section | -0.038(-0.235; 0.16) | 0.708 | 33.3% | -37.1% | 33.33% \| 66.67% | 31.44% \| 68.56% |
|  | **Macrosomia** |  |  |  |  |  |
| Pre-pregnancy weight (kg) | 0.538(0.33; 0.746) | <0.001 | 13.4% | 40.4% | 43.3% \| 56.7% | 70.2% \| 29.8% |
| BMI (c.) | 0.506(0.298; 0.715) | <0.001 | 3.1% | 47.5% | 48.45% \| 51.55% | 73.77% \| 26.23% |
| Pre-pregnancy BMI (kg/m²) | 0.499(0.291; 0.708) | <0.001 | 9.3% | 40.7% | 45.36% \| 54.64% | 70.33% \| 29.67% |
| GWG above the range | 0.499(0.293; 0.706) | <0.001 | 19.6% | 30.3% | 40.21% \| 59.79% | 65.17% \| 34.83% |
| BMI ≥ 25 kg/m² | 0.488(0.28; 0.697) | <0.001 | 1.0% | 47.8% | 49.48% \| 50.52% | 73.91% \| 26.09% |
| Maternal height (cm) | 0.452(0.248; 0.656) | <0.001 | 27.8% | 17.4% | 36.08% \| 63.92% | 58.68% \| 41.32% |
| Fetal sex: Son | 0.377(0.18; 0.575) | <0.001 | 38.1% | -0.4% | 30.93% \| 69.07% | 49.8% \| 50.2% |
| BMI ≥ 30 kg/m² | 0.31(0.136; 0.484) | <0.001 | -52.6% | 83.6% | 76.29% \| 23.71% | 91.79% \| 8.21% |
| Maternal height > 170 cm | 0.31(0.109; 0.512) | 0.002 | -25.8% | 56.8% | 62.89% \| 37.11% | 78.41% \| 21.59% |
| Maternal height > 160 | 0.214(0.086; 0.343) | 0.001 | 81.4% | -60.0% | 9.28% \| 90.72% | 20% \| 80% |
| Prior macrosomia | 0.159(-0.038; 0.356) | 0.114 | -34.0% | 49.9% | 67.01% \| 32.99% | 74.97% \| 25.03% |
| Gestational age ≥ 38 weeks | 0.155(0.073; 0.237) | <0.001 | 93.8% | -78.3% | 3.09% \| 96.91% | 10.86% \| 89.14% |
| Family: diabetes in the mother | 0.152(-0.051; 0.355) | 0.143 | -25.8% | 40.9% | 62.89% \| 37.11% | 70.46% \| 29.54% |
| Family: diabetes in the father | 0.152(-0.051; 0.355) | 0.143 | -25.8% | 40.9% | 62.89% \| 37.11% | 70.46% \| 29.54% |
| Folic acid supplementation | 0.119(-0.09; 0.328) | 0.264 | -13.4% | 25.3% | 56.7% \| 43.3% | 62.65% \| 37.35% |
| Prior diabetes | 0.117(-0.055; 0.29) | 0.183 | 58.8% | -47.0% | 20.62% \| 79.38% | 26.49% \| 73.51% |
| GDM | 0.115(-0.057; 0.287) | 0.190 | -56.7% | 68.2% | 78.35% \| 21.65% | 84.11% \| 15.89% |
| Prior cesarean section | 0.114(-0.086; 0.313) | 0.264 | -32.0% | 43.3% | 65.98% \| 34.02% | 71.66% \| 28.34% |
| Village | 0.086(-0.111; 0.282) | 0.393 | -36.1% | 44.6% | 68.04% \| 31.96% | 72.32% \| 27.68% |
| Education < 12 years | 0.056(-0.065; 0.176) | 0.365 | -81.4% | 87.0% | 90.72% \| 9.28% | 93.51% \| 6.49% |
| Interpregnancy interval (c.) | 0.048(-0.158; 0.253) | 0.649 | 23.7% | -18.9% | 38.14% \| 61.86% | 40.53% \| 59.47% |
| Ex-smoking | 0.042(-0.105; 0.19) | 0.574 | -71.1% | 75.4% | 85.57% \| 14.43% | 87.68% \| 12.32% |
| Lower financial status | 0.036(-0.116; 0.188) | 0.639 | -69.1% | 72.7% | 84.54% \| 15.46% | 86.36% \| 13.64% |
| Marital status: married | 0.03(-0.157; 0.217) | 0.752 | -46.4% | 49.4% | 73.2% \| 26.8% | 74.7% \| 25.3% |
| Multivitamin supplementation | 0.028(-0.18; 0.235) | 0.794 | 19.6% | -16.8% | 40.21% \| 59.79% | 41.59% \| 58.41% |
| Never smoking | 0.012(-0.149; 0.173) | 0.880 | 64.9% | -63.7% | 17.53% \| 82.47% | 18.15% \| 81.85% |

* NRI (95%CI): Net Reclassification Improvement (95% confidence intervals); ** *p*-Value <0.05 was statistically significant; *** Base model: maternal age + parity categories (i.e. 0, 1, 2 and ≥3 deliveries). LGA: birth weight > 90th percentile (analysis for 99 cases vs. 741 newborns 10−90th percentile); Macrosomia: birth weight > 4000 g (analysis for 97 cases vs. 755 newborns 2500−4000 g); BMI: body mass index; GWG: gestational weight gain; GDM: gestational diabetes mellitus. The comments:

Down: a decrease in the likelihood of disease. Up: increase in the likelihood of disease.

An example for the model with pre-pregnancy weight (kg) extension for LGA prediction:

The NRI (1) is an assessment for the sick participants, and it represents the difference in the value of the increase (56.57%) and the decrease (43.43%) in probability in the model after adding a factor. NRI (1) = 13.1% (56.57% - 43.43%) means that in 13.1% of ill patients there was a "correct" reclassification in the direction of increased disease probability after adding pre-pregnancy weight (kg) to the model.

The NRI (0) is an assessment for the healthy participants, and it represents the difference in the value of the increase (30.63%) and the decrease (69.37%) in probability in the model after adding a factor. NRI (0) = 38.7% (69.37% - 30.63%) means that in 38.7% of the healthy persons there was a "correct" reclassification towards a decrease in the probability of the disease after adding pre-pregnancy weight (kg) to the model.
