# Supplementary material for: The Role of Maternal Weight in the Hierarchy of Macrosomia Predictors; Overall Effect of Analysis of Three Prediction Indicators
Source: Nutrients. 2021 Feb 28;13(3):801. doi: 10.3390/nu13030801 (PMC8000437; doi:10.3390/nu13030801)
Supplement: Supplementary file 1 [file nutrients-13-00801-s001.zip › Table S6.docx]

**Table S6.** Values of the three predictive indicators (AUC, IDI, NRI) in the extended multivariate models for the probability of LGA and macrosomia.

|  | |  | | |  | | **LGA** | | | |  | |  |  | |
| --- | --- | --- | --- | --- | --- | --- | --- | --- | --- | --- | --- | --- | --- | --- | --- |
| Base model  (maternal age + parity c*)  **Extended models**  **(base model + listed variables)** | | AUC base  0.574  **AUC extended** | | | | P **  (extended  vs base) | | | **IDI** | | P **  (extended  vs base) | | **NRI** | P **  (extended  vs base) | |
| Pre-pregnancy BMI (kg/m²) | | 0.669 | | | | <0.001 | | | 0.031 | | <0.001 | | 0.459 | <0.001 | |
| BMI (c.) | | 0.661 | | | | <0.001 | | | 0.028 | | <0.001 | | 0.431 | <0.001 | |
| BMI ≥ 25 kg/m² | | 0.645 | | | | <0.001 | | | 0.018 | | 0.001 | | 0.407 | <0.001 | |
| BMI ≥ 30 kg/m² | | 0.637 | | | | <0.001 | | | 0.024 | | 0.001 | | 0.312 | <0.001 | |
| Pre-pregnancy weight (kg) | | 0.694 | | | | <0.001 | | | 0.044 | | <0.001 | | 0.519 | <0.001 | |
| GWG above the range | | 0.655 | | | | <0.001 | | | 0.025 | | <0.001 | | 0.455 | <0.001 | |
| Gestational age ≥ 38 weeks | | 0.595 | | | | 0.002 | | | 0.005 | | 0.024 | | 0.131 | 0.022 | |
| Prior diabetes | | 0.575 | | | | 0.015 | | | 0.0002 | | 0.636 | | 0.171 | 0.042 | |
| Prior cesarean section | | 0.575 | | | | 0.016 | | | 0.00001 | | 0.917 | | -0.038 | 0.708 | |
| Prior macrosomia | | 0.587 | | | | 0.005 | | | 0.025 | | 0.008 | | 0.043 | 0.648 | |
| Family: diabetes in the mother | | 0.591 | | | | 0.003 | | | 0.004 | | 0.107 | | 0.182 | 0.078 | |
| Family: diabetes in the father | | 0.591 | | | | 0.003 | | | 0.004 | | 0.104 | | 0.182 | 0.078 | |
| Folic acid supplementation | | 0.577 | | | | 0.013 | | | 0.0001 | | 0.877 | | 0.071 | 0.497 | |
| Multivitamin supplementation | | 0.578 | | | | 0.012 | | | 0.001 | | 0.280 | | 0.08 | 0.444 | |
| Maternal height (cm) | | 0.629 | | | | <0.001 | | | 0.013 | | 0.002 | | 0.35 | 0.001 | |
| Maternal height > 160 cm | | 0.608 | | | | <0.001 | | | 0.008 | | 0.002 | | 0.21 | 0.001 | |
| Maternal height > 170 cm | | 0.621 | | | | <0.001 | | | 0.012 | | 0.006 | | 0.293 | 0.004 | |
| Never smoking | | 0.576 | | | | 0.013 | | | 0.0004 | | 0.365 | | 0.033 | 0.676 | |
| Ex-smoking | | 0.583 | | | | 0.007 | | | 0.0004 | | 0.696 | | 0.057 | 0.450 | |
| Education < 12 years | | 0.574 | | | | 0.017 | | | -0.000001 | | 0.986 | | 0.021 | 0.713 | |
| Village | | 0.581 | | | | 0.008 | | | 0.002 | | 0.240 | | 0.112 | 0.268 | |
| Lower financial status | | 0.574 | | | | 0.017 | | | 0.000009 | | 0.496 | | -0.005 | 0.948 | |
| Marital status: married | | 0.579 | | | | 0.011 | | | 0.0006 | | 0.526 | | 0.094 | 0.287 | |
| Interpregnancy interval (c.) | | 0.594 | | | | 0.002 | | | 0.005 | | 0.061 | | 0.110 | 0.302 | |
| Fetal sex: Son | | 0.569 | | | | 0.026 | | | 0.0005 | | 0.494 | | 0.065 | 0.540 | |
| GDM | | 0.586 | | | | 0.005 | | | 0.01 | | 0.016 | | 0.226 | 0.014 | |
|  |  | | |  | | | | **Macrosomia** | | | |  |  |  | |
| Base model  (maternal age + parity c*)  **Extended models**  **(base model + listed variables)** | | | AUC base  0.564  **AUC extended** | | | P **  (extended) | | | **IDI** | P **  (extended  vs base) | | | **NRI** | | P **  (extended  vs base) |
| Pre−pregnancy BMI (kg/m²) | | | 0.671 | | | <0.001 | | | 0.041 | <0.001 | | | 0.499 | | <0.001 |
| BMI (c.) | | | 0.666 | | | <0.001 | | | 0.036 | <0.001 | | | 0.506 | | <0.001 |
| BMI ≥ 25 kg/m² | | | 0.653 | | | <0.001 | | | 0.028 | <0.001 | | | 0.488 | | <0.001 |
| BMI ≥ 30 kg/m² | | | 0.612 | | | <0.001 | | | 0.026 | 0.001 | | | 0.31 | | <0.001 |
| Pre−pregnancy weight (kg) | | | 0.706 | | | <0.001 | | | 0.061 | <0.001 | | | 0.538 | | <0.001 |
| GWG above the range | | | 0.656 | | | <0.001 | | | 0.029 | <0.001 | | | 0.499 | | <0.001 |
| Gestational age ≥ 38 weeks | | | 0.602 | | | 0.001 | | | 0.009 | <0.001 | | | 0.155 | | <0.001 |
| Prior diabetes | | | 0.571 | | | 0.022 | | | 0.002 | 0.001 | | | 0.117 | | 0.183 |
| Prior cesarean section | | | 0.568 | | | 0.029 | | | 0.0006 | 0.515 | | | 0.114 | | 0.264 |
| Prior macrosomia | | | 0.611 | | | <0.001 | | | 0.044 | 0.001 | | | 0.159 | | 0.114 |
| Family: diabetes in the mother | | | 0.588 | | | 0.005 | | | 0.002 | 0.205 | | | 0.152 | | 0.143 |
| Family: diabetes in the father | | | 0.584 | | | 0.007 | | | 0.003 | 0.138 | | | 0.152 | | 0.143 |
| Folic acid supplementation | | | 0.570 | | | 0.025 | | | 0.002 | 0.286 | | | 0.119 | | 0.264 |
| Multivitamin supplementation | | | 0.567 | | | 0.032 | | | 0.0003 | 0.655 | | | 0.028 | | 0.794 |
| Maternal height (cm) | | | 0.651 | | | <0.001 | | | 0.023 | <0.001 | | | 0.452 | | <0.001 |
| Maternal height > 160 cm | | | 0.602 | | | 0.001 | | | 0.008 | 0.004 | | | 0.214 | | 0.001 |
| Maternal height > 170 cm | | | 0.618 | | | <0.001 | | | 0.014 | 0.003 | | | 0.31 | | 0.002 |
| Never smoking | | | 0.564 | | | 0.040 | | | 0.0001 | 0.663 | | | 0.012 | | 0.880 |
| Ex−smoking | | | 0.576 | | | 0.015 | | | 0.0002 | 0.834 | | | 0.042 | | 0.574 |
| Education < 12 years | | | 0.570 | | | 0.024 | | | 0.001 | 0.606 | | | 0.056 | | 0.365 |
| Village | | | 0.568 | | | 0.028 | | | 0.001 | 0.317 | | | 0.086 | | 0.393 |
| Lower financial status | | | 0.571 | | | 0.023 | | | 0.0002 | 0.736 | | | 0.036 | | 0.639 |
| Marital status: married | | | 0.569 | | | 0.027 | | | 0.0004 | 0.544 | | | 0.03 | | 0.752 |
| Interpregnancy interval (c.) | | | 0.588 | | | 0.005 | | | 0.004 | 0.080 | | | 0.048 | | 0.649 |
| Fetal sex: Son | | | 0.626 | | | <0.001 | | | 0.015 | <0.001 | | | 0.377 | | <0.001 |
| GDM | | | 0.573 | | | 0.019 | | | 0.002 | 0.195 | | | 0.115 | | 0.190 |
|  | | |  | | |  | | |  |  | | |  | |  |

* parity (c) categories: 0, 1, 2 and ≥3 deliveries; * P−value <0.05 was statistically significant. LGA: birth weight > 90th percentile (analysis for 99 cases vs. 741 newborns 10−90th percentile); Macrosomia: birth weight > 4000 g (analysis for 97 cases vs. 755 newborns 2500−4000 g); AUC: area under receiver operating characteristic curve; IDI: Integrated Discrimination Improvement; NRI: Net Reclassification Improvement; BMI: body mass index; GWG: gestational weight gain; GDM: gestational diabetes mellitus.
